# Single-cell study of human ovarian response to phthalate exposure reveals glial cell susceptibility and disruption of adhesion and mitochondrial pathways

**DOI:** 10.1016/j.ebiom.2026.106487

**Published:** 2026-10-06

**Authors:** Eleftheria Maria Panagiotou, Loren Méar, Ilmatar Rooda, Alesandro Haxhiu, Elisabeth Moussaud-Lamodière, Karolina Wasilewska, Tianyi Li, Catarina Arnelo, Karin Pettersson, Kiriaki Papaikonomou, Cecilia Lindskog, Pauliina Damdimopoulou

**Affiliations:** a Department of Women's and Children's Health, Karolinska Institutet, Stockholm, Sweden; b Department of Gynecology and Reproductive Medicine, Karolinska University Hospital, Stockholm, Sweden; c Department of Immunology, Genetics and Pathology, Cancer Precision Medicine, Uppsala University, Uppsala, Sweden; d Department of Clinical Science, Intervention and Technology, Karolinska Institutet, Stockholm, Sweden; e Department of Oncology-Pathology, Karolinska Institutet, Stockholm, Sweden; f Department of Pregnancy and Delivery, Karolinska University Hospital, Stockholm, Sweden

**Keywords:** Phthalates, Human ovary, Tissue explant culture, Female fertility, Endocrine disrupting chemicals, Single cell RNA sequencing

## Abstract

**Background:**

Phthalates are known male endocrine disruptors and reproductive toxicants. Despite growing evidence of female effects, mechanistic knowledge in key reproductive organs remains limited, hindering regulations. Here, we conducted an experimental study to map the impact of mono(2-ethylhexyl) phthalate (MEHP), the primary metabolite of di(2-ethylhexyl) phthalate (DEHP), on adult human ovarian tissue at single-cell resolution.

**Methods:**

Ovarian tissue explants from seven donors (five gender-affirming surgery, two caesarean-section) were exposed to two MEHP concentrations: an epidemiologically relevant 20.51 nM and a 1000-fold higher 20.51 μM. After six days, single-cell RNA sequencing (one donor) and immunostainings (six donors) were used to characterise gene expression changes across cell types. Primary ovarian cells derived from three gender-affirming surgery patients and stem cell-derived Schwann cells were used for validation.

**Findings:**

Explants retained all major ovarian cell types, including rare glial cells and oocytes. MEHP altered transcriptomes across all cell types, disrupting pathways related to actin cytoskeleton, cell adhesion, and oxidative phosphorylation (OXPHOS). Protein analyses confirmed altered *EIF5A*, *MT-ND3*, *MT-ND4L*, and *VCL* expression, genes linked to mitochondrial translation, OXPHOS, adhesion, and cytoskeleton. MEHP also reduced cell-cell communication, particularly glial-stromal interactions. Mitochondrial stress assay in primary cells indicated increased proton leakage, and Schwann cell responses mirrored the scRNA-seq findings.

**Interpretation:**

Ovarian toxicity studies traditionally focus on follicles, but this study reveals the susceptibility of non-follicular cells. Importantly, the sensitivity of ovarian nervous system cells to MEHP offers new mechanistic insights into reported links between phthalates, altered ovarian sensitivity index, and polyendocrine metabolic ovarian syndrome. Our findings highlight the need for a holistic assessment of ovarian toxicity that considers all ovarian cell types, not only follicles.

**Funding:**

Swedish Research Council for Sustainable Development and the European Union.

**Research in context**

**Evidence before this study**

DEHP is an endocrine-disrupting reproductive toxicant known to lower male fertility by reducing androgen levels. More recently, DEHP has been associated with adverse effects on female reproduction, including disruption of folliculogenesis and steroidogenesis in rodents and in follicle culture studies. Our previous work demonstrated that the DEHP metabolite MEHP disrupts growth and viability of human cortical ovarian follicles in vitro, and associates with lower ovarian sensitivity index in patients undergoing infertility treatment.

**Added value of this study**

This study delineates phthalate toxicity in human ovaries with cell type precision. By integrating single-cell RNA sequencing with immunofluorescence validation in MEHP-exposed ovarian explants, we demonstrate disruption of oxidative phosphorylation, cell adhesion and cytoskeletal pathways across all cell types. Surprisingly, many somatic cells, such as glial cells, showed greater disruption than the follicles, which are typically the main focus of ovarian studies.

**Implications of all the available evidence**

Single-cell technologies have made the diversity of somatic cell types in human ovaries evident, and our study demonstrates that non-follicular cells are also targets of chemical disruption. Taken together, current evidence indicates that phthalates disrupt human ovarian function at epidemiologically relevant concentrations, extending beyond the follicular compartment. In particular, the observed susceptibility of glial cells to MEHP offers new modes of action for environmental chemical mediated disruption of ovarian biology and may explain reported links between phthalate exposure and polycystic ovaries. These findings should encourage ovarian toxicity research to expand its focus beyond follicles and guide chemical safety testing accordingly.

## Introduction

Phthalates constitute a group of chemicals known for their endocrine-disrupting properties and adverse effects on fertility in both sexes.^1^^,^^2^ They are commonly used as plasticisers and solvents in various everyday products such as lotions, perfumes, and cosmetics.^3^ Due to their non-covalent binding in plastics, phthalates can leach into the environment, contaminating food, water, soil, air, and dust, thereby becoming widespread environmental pollutants.^3^ Furthermore, as plastic additives, phthalates are released into the environment and expose biota through micro- and nanoplastic pollution, contributing to human disease.^4^ For example, a recent report estimated that di(2-ethylhexyl) phthalate (DEHP), a phthalate used in soft plastics, contributes to an increased risk of obesity, type 2 diabetes, endometriosis, and cardiovascular mortality, with an estimated cost of 320 million USD per year in the US.^5^

Human exposure to phthalates is pervasive, with detectable levels found in tissues like the liver, lungs, and adipose tissue, as well as in biological fluids such as serum, urine, ovarian follicular fluid, and amniotic fluid.^6^, ^7^, ^8^ Their presence in amniotic fluid suggests that exposure begins as early as in utero.^6^ Despite their rapid metabolism and excretion, phthalates are considered “semi-persistent” due to continuous human exposure and high absorption rates.^6^ Notably, phthalate levels in follicular fluid correlate with personal care product use, underscoring the influence of lifestyle choices on exposure patterns.^9^

Human epidemiological studies have found correlations between urinary phthalate levels and various reproductive challenges in women, including infertility, prolonged time to conception, early onset of menopause, premature ovarian failure, reduced antral follicle counts, and diminished ovarian responsiveness to gonadotropin stimulation during assisted reproductive technologies.^8^^,^^10^, ^11^, ^12^, ^13^, ^14^, ^15^, ^16^, ^17^ Given the finite number of follicles, the potential effects of phthalates on the ovary can substantially impact female fertility. A range of in vitro and rodent in vivo studies have demonstrated the negative effects of phthalates on both main ovarian functions, folliculogenesis and steroidogenesis.^18^^,^^19^ Despite existing evidence of adverse effects of phthalates on female reproduction in experimental models, their mechanisms of action remain understudied in humans, posing challenges for chemical health risk assessment. For example, the current risk assessment for the phthalate DEHP is based on male reproductive health outcomes, although our recent evaluation of animal study data suggests that females are at least as sensitive as males.^20^ As women of reproductive age exhibit higher phthalate exposure levels than men, it is crucial to investigate phthalate effects on female reproduction using human-relevant models.^21^

We have previously demonstrated that one-week exposure of human ovarian tissue to the DEHP metabolite mono(2-ethylhexyl) phthalate (MEHP) in culture disrupts follicular development and survival.^22^ Bulk transcriptomic analyses of ovarian cell lines and primary cells further suggested the dysregulation of cytoskeletal organisation and the Hippo signalling pathway as potential mechanisms underlying these effects.^22^ However, since our transcriptomic analyses were carried out in exposed 2D cell cultures, the study could not provide insights into the native target cell types within human ovaries. The ovary is a highly heterogeneous organ, consisting of germ cells (oocytes) and various somatic cell types, including granulosa, stromal, immune, perivascular, endothelial, epithelial, and glial cells.^23^^,^^24^ Despite the well-acknowledged cellular diversity of the ovary, environmental chemical toxicity studies have focused almost exclusively on follicles, leaving the sensitivity of most ovarian cell types to environmental stressors largely unexplored.^25^ A comprehensive understanding of cellular responses to chemical stressors is essential; without this knowledge, ovarian toxicity cannot be accurately characterised, predicted, or prevented. Therefore, in this study, we aimed to map the effects of MEHP on human ovarian tissue at single-cell resolution to delineate the responses across cell types and identify critically sensitive cell populations.

## Methods

### Chemicals and concentration selection

MEHP (CAS number: 4376-20-9) was provided in a 1 M quality-controlled stock solution in Dimethyl sulfoxide (DMSO, CAS number: 67–68–5, >98.9% HPLC, Sigma Aldrich, Stockholm, Sweden) by Prof. Christian Lindh (Division of Occupational and Environmental Medicine, Lund University, Sweden).

Concentrations of MEHP were selected based on the Swedish Environmental Longitudinal, Mother and child, Asthma and allergy (SELMA) cohort study as previously described.^26^ In this study, 1X MEHP denotes the estimated serum mean of exposure levels in SELMA pregnant individuals (20.51 nM), as previously described.^22^ The 1000X condition represents a 1000-fold higher concentration (20.51 μM).

The 1 M MEHP stock solution was diluted to a 1 mM working stock, which was validated using liquid chromatography coupled with tandem mass spectrometry (LC-MS/MS) at the Finnish Institute for Health and Welfare and found to have concentration of 1.58 mM. This stock was further diluted in DMSO to prepare experiment stocks that produced the desired nominal exposure concentrations when diluted 1000-fold in culture medium. The final DMSO concentration was 0.1% in all samples.

### Ethics

Patients were informed about the project, given access to its website and provided with contact details for further enquiries. Prior to any sampling, signed informed consents were obtained from all participants in accordance with the Declaration of Helsinki. Participants consented to the use of ovarian tissue for research, collection of personal information, and biobanking. The study was approved by the Swedish Ethical Review Authority (licence number 2015–798/31 and 2024-08606 with amendments), and all biological samples were registered with a biobank in compliance with the Swedish Biobanks in Medical Care Act. Samples and data were pseudonymised, and data were securely stored in encrypted online systems, accessible only to authorised personnel. Table 1 provides baseline characteristics of study participants and details of the experimental methods used. Human ovarian tissue samples were collected from October 2022 to March 2026, and experimental procedures and data collection were conducted from October 2022 to May 2026.

**Table 1:** Clinical characteristics of study participants and experimental sample allocation.

Donor^a^	Type of operation	Age	BMI	Smoking status	Experiments		
					scRNA-seq	Staining	Seahorse
1	gender-affirming	23	29.4	non-smoker	x		x
2		26	29.8	non-smoker		x	
3		32	26	non-smoker		x	
4		25	31.5	not for 6 years		x	
5		33	25.3	not for 2 years		x	
6		27	19.8	not for 6 months			x
7		25	29.4	non-smoker			x
8	c-section	33	30	non-smoker		x	
9		33	27	non-smoker		x	

a None of the donors had a history of diagnosed ovarian disease.

### Ovarian tissue collection

Ovarian tissue was collected from nine consenting individuals at Karolinska University Hospital Huddinge, Sweden, including seven gender-affirming patients undergoing hysterectomy and bilateral salpingo-oophorectomy and two individuals undergoing elective caesarean section (c-section). A human ovarian cortical tissue explant culture model was employed to assess the cell-type specific effects of MEHP on gene expression, as previously described.^27^ Of the nine donors, seven contributed tissue to explant cultures. Explant-cultured tissue was used for single-cell RNA sequencing (n = 1, gender-affirming donor) and immunofluorescence staining (n = 6, four gender-affirming donors and two c-section donors). Tissue from three gender-affirming donors was used for primary cell cultures for mitochondria stress assays; one donor contributed to both the explant culture and primary cell culture experiments. In total, the study included tissue from nine unique donors (Table 1).

Assessment of patient records indicated that none of the tissue donors had a history of diagnosed ovarian disease, and none were current smokers. All gender-affirming surgery patients had received androgen therapy for 3–8 years prior to biopsy. Inclusion of samples from different patient groups ensured sufficient material for the study and increased the generalisability of the findings. Our previous research has demonstrated that cortical tissues from these patient groups have comparable cell compositions and respond to MEHP in a similar manner.^22^^,^^28^

### Ovarian tissue explant culture

All samples were collected directly from the operating theatre in Dulbecco's Phosphate Buffered Saline containing calcium, magnesium chloride, glucose, and pyruvate (Life Technologies, Grand Island, USA), and were placed in culture within 45 min in an air-liquid interphase on laminin-coated (LN221, BioLamina, Sundbyberg, Sweden) cell culture inserts (PIHP01250, Merck Millipore, Darmstadt, Germany), as previously published.^27^ Tissue received from gender-affirming surgeries consisted of both cortex and medulla, whereas the c-section samples contained cortex only. Only cortical tissue was cultured. The samples were processed into 1 mm^3^ cortical explants, which were distributed among all culture groups (DMSO, 1X MEHP, and 1000X MEHP) and cultured one explant per well.

Tissue was exposed for six days, with complete media changes and re-exposure every 48 h. A vehicle control (0.1% DMSO) was also included. On day six, explants from one patient (donor 1, 23 years old) were dissociated into single cells for library preparation as described below, and samples from the other six patients were fixed in 4% paraformaldehyde (Thermo Scientific, Rockford, USA) for immunostaining validation experiments.

### Single-cell sequencing of MEHP exposed ovarian explants

#### Library preparation and sequencing

Cultured ovarian tissue was dissociated to single cells using a combination of enzymatic and mechanic dissociation, as previously described.^28^ Following dissociation, cells were counted, and viability was assessed to ensure that a high proportion of viable cells (>90% for all samples) proceeded to downstream processing. Three single-cell RNA-sequencing libraries (DMSO, 1X MEHP, 1000X MEHP) were prepared using Chromium Next GEM Single Cell 3’ Reagent Kits v3.1 (10x genomics) according to the manufacturer's instructions with estimated 9584, 9603, and 9507 cells loaded per library, respectively. After cDNA amplification, the samples were sent for quality control to the core facility for Bioinformatics and Expression Analysis (BEA) at Karolinska Institutet, using High Sensitivity D5000 ScreenTape (Agilent Technologies, Santa Clara, USA). The constructed libraries were then sequenced at the same facility using NextSeq2000 platform. Raw data were demultiplexed with the cellranger (version 6·1·1) *mkfastq* function and aligned to the human reference genome GRCh38 using the *cellranger count* function.

#### General data analysis

The three filtered count datasets generated by CellRanger were analysed using the Seurat package (version 5·1·0) in R (version 4·3·2). These corresponded to the two MEHP concentrations (1X and 1000X) and the vehicle control (DMSO), with initial recovered cell counts of 3652, 4847, and 4267 cells, respectively. Filtering steps were applied independently to each dataset to remove cells with fewer than 200 features, more than 5000 features, or mitochondrial gene expression exceeding 30%. After quality control, 12,005 cells remained for analysis (3347 cells from 1X MEHP, 4710 from 1000X MEHP, and 3948 from the DMSO control). Data processing followed the standard Seurat workflow, including normalisation, scaling, and principal component analysis (PCA) for each dataset independently.

The datasets were integrated using the reciprocal PCA (RPCA) method, resulting in the identification of 18 distinct cell clusters. Each cluster was assigned an identity based on canonical marker expression, as determined from the literature and the Human Protein Atlas (HPA) knowledge resource.^24^^,^^28^^,^^29^ The clusters were named according to the predominant cell type: oocytes (*ZP3*, *OOSP2* and *FIGLA*), granulosa cells (*BEX1*, *FOXL2*, and *AMHR2*), stromal cells (*DCN*, *LUM*, and *COL3A1*), perivascular cells (*ACTA2*, *MYH11*, and *TAGLN*), endothelial cells (*VWF*, *CDH5* and *CD34*), immune cells (*CD68*, *CD4*, and *CXCR4*), and glial cells (*MPZ*, *SOX10*, *S100B*) (Supplementary File S1). To further confirm cell identities, differential gene expression analysis was performed for each cell population compared to all others using Seurat's FindAllMarkers function and the Wilcoxon rank-sum test (unadjusted p < 0.05, and log2FC > 2) to determine expression signatures. The significantly upregulated genes from these signatures were subsequently analysed for Gene Ontology (GO) enrichment in the biological processes category using the clusterProfiler R package (version 4·10·1).

#### Analysis of MEHP effects on ovarian cortical cell populations

Differential gene expression analysis was performed for each cell population using a significance threshold of unadjusted p < 0.05 and a log_2_ fold-change cutoff of 0.5, across the following comparisons: DMSO vs. 1X MEHP and DMSO vs. 1000X MEHP. As environmentally relevant contaminant exposures are not expected to induce overt toxicity or dramatic transcriptomic alterations, unadjusted p-values combined with fold-change filtering were used to capture subtle but biologically coordinated responses. The FindMarkers function in Seurat was used to identify both upregulated and downregulated genes. GO enrichment analyses were conducted on the resulting DEG lists to identify biological processes affected by MEHP exposure across the three GO categories: biological processes, cellular components, and molecular functions. Additionally, cell–cell communication analyses for the DMSO vs. 1X MEHP and DMSO vs. 1000X MEHP comparisons were performed using the CellChat package (version 2·1·2),^30^ based on the Secreted Signalling, ECM-receptor, and Cell–cell contact reference databases. Metabolic scores were predicted using scMetabolism package (version 0·2·1) on the count matrix against the Kyoto Encyclopedia of Genes and Genomes (KEGG) dataset using AUCell method,^31^ with associated statistics was performed using Kruskal–Wallis with Dunn's test followed by Benjamini–Hochberg correction for multiple comparison.

To predict upstream ligand-receptor interactions driving transcriptional changes in ovarian glial cells, NicheNet analysis was performed using the nichenetr R package (version 2·2·1·1).^32^ Glial cells were defined as the receiver cell population, with all remaining ovarian cell types, granulosa, stromal, perivascular, endothelial, immune and oocyte cells designated as sender populations. The NicheNet prior model databases (ligand-target matrix, ligand-receptor network and weighted signalling networks) were obtained from Zenodo. Potential ligands were defined as those expressed in at least one sender cell type and interacting with receptors expressed in glial cells. Ligand activity was scored using the area under the precision–recall curve (AUPR) against the full glial cell DEG lists for each comparison (DMSO vs. 1X MEHP: n = 231 genes; DMSO vs. 1000X MEHP: n = 192 genes), identifying the top 15 predicted upstream ligands per comparison. To analyse predicted ligand activity, differential expression of top-ranked ligands was subsequently examined across all sender cell populations using the DEG results from the DMSO vs. 1X MEHP and DMSO vs. 1000X MEHP comparisons, applying the same significance threshold of p < 0.05.

#### Validation of single-cell RNA-sequencing findings by immunostaining of ovarian tissue sections

Target genes for immunostaining validation were selected based on the following criteria: i) differential expression in response to MEHP, ii) association with cell adhesion, oxidative phosphorylation or cytoskeleton based on GO terms or the literature, and iii) availability of validated high-reliability antibodies in the HPA database. Two immunostaining approaches were used: immunofluorescence staining, in which antibodies from different species can be combined in the same run, and a sequential immunohistochemistry approach using OPAL fluorophores, enabling multiplexing of antibodies from the same species.

For immunofluorescent validation, *VCL* (vinculin) was selected for its association with multiple cell adhesion related GOs in oocyte and stromal cells, *EIF5A* (eukaryotic translation initiation factor 5A) due to its translational roles related to mitochondria and response to phthalates,^22^ and *MT-ND3* (mitochondrially encoded NADH:ubiquinone oxidoreductase core subunit 3) and *MT-ND4L* (mitochondrially encoded NADH:ubiquinone oxidoreductase core subunit 4L) as complex I genes affected across most cell types (Supplementary File S2). Their expression levels were assessed by immunofluorescence staining in MEHP-exposed ovarian cortical tissue obtained from an independent set of six patients cultured under identical exposure conditions (Table 1). To enable cell type-specific signal quantification across main ovarian cell types, tissues were co-stained with either a glial cell marker MPZ^33^ or an immune cell marker PTPRC.^34^ Other structures, such as blood vessels and follicles, were identified based on their distinct morphology.

Sections of paraffin-embedded tissues of 4 μm thickness were prepared from all six patients. After deparaffinization and rehydration through xylene and a graded ethanol series, antigen retrieval was performed at 96°C in Tris-buffered saline/EDTA (Sigma Aldrich, St. Louis, USA). Sections were then incubated for 1 h at room temperature in a blocking solution containing 5% (w/v) bovine serum albumin, 20% (v/v) normal donkey serum, and Tris-buffered saline, followed by overnight incubation at 4°C with primary antibodies against the selected targets (antibody names, catalogue numbers, suppliers and RRIDs are provided in Supplementary Table S1). Secondary antibody incubation was performed for 1 h at room temperature (Supplementary Table S1). Nuclei were counterstained with DAPI (Thermo Scientific, Rockford, USA, 1:1000 dilution), and slides were mounted using ProLong Gold antifade reagent (Thermo Scientific, Rockford, USA). Secondary-only controls (samples incubated with either secondary antibody but without primary antibodies to assess non-specific secondary binding), negative controls (no antibodies to assess tissue autofluorescence), and isotype controls (using rabbit IgG to assess non-specific staining) were included. Sections were imaged using a widefield inverted microscope system (Nikon Eclipse Ti, Tokyo, Japan) in the Live cell imaging (LCI) facility at Karolinska Institutet. Images were acquired using 20X/0.75 air objective.

Fluorescence signals were quantified using CellProfiler 4.2.6.^35^ All images first underwent illumination correction. Histologically distinct structures (follicles, vessels) were then manually segmented to enable quantification of mean fluorescent signals from different structures. Specifically, follicles and oocytes were segmented, with oocytes serving as masks to segment the surrounding follicular cells. Fluorescence data were collected from individual oocytes and follicular cell groups within each follicle. For vessels, vascular cells were identified as the inner monolayer adjacent to the lumen, and fluorescence signals were quantified from the entire vascular cell layer in each vessel. For stromal tissue, images containing follicles, but no visible blood vessels were selected. The total tissue area was defined, follicles were masked, and fluorescent signals were quantified from the remaining area, with each section treated as a single data point. For the rare cell types immune cells and glial cells, co-staining with cell type-specific markers (PTPRC and MPZ, respectively) enabled automated segmentation, with each data point representing a positive signal area corresponding to that cell type. Number of datapoints used per cell type per target can be found in Supplementary Table S2.

Immunofluorescence data were tested for normality using the Shapiro–Wilk test, followed by the Kruskal–Wallis test with Dunn's post hoc comparison. All conditions were compared to the vehicle control, and statistical significance was defined as p < 0.05.

Additional sequential immunohistochemical staining focussing on glial cells was performed on tissue sections from four patients, as previously described.^36^ *FBLN2* (fibulin 2) was selected as a glial cell-specific MEHP-regulated target gene associated with cell adhesion (Supplementary File S2). Markers for Schwann cells (MPZ) and endothelial cells (CD34) were added for orientation. Briefly, slides underwent a three-cycle multiplex immunostaining protocol with antibody stripping/deactivation steps performed after each cycle by heating at 90°C for 20 min in 1 × Target Retrieval Solution, pH 6.0 (Agilent Technologies). Three tyramide signal amplification (TSA)-linked OPAL fluorophores (OPAL 520, OPAL 570, and TSA-DIG/780; Akoya Biosciences, Marlborough, MA, USA) were used. Fluorophore-antibody pairings were selected according to expected marker co-expression and relative abundance to minimise spectral crosstalk and optimise signal intensity. The following primary antibodies from HPA were used: CD34 for endothelial cells (CAB000018, 1:900, RRID: AB_2063006, TSA-DIG/780), MPZ for Schwann cells (HPA068925, 1:2000, RRID: AB_2732123, OPAL 570), and FBLN2 as the candidate marker (HPA001934, 1:250, RRID: AB_1078827, OPAL 520).

Whole-slide multispectral images were acquired using the Akoya PhenoImager with a 40 × objective. Digital qptiff images were generated for downstream quantitative and spatial analyses. Multiplex immunohistochemistry images were analysed using QuPath (v0.6.0). For each condition, one representative tissue region was manually annotated as the region of interest (ROI). Marker positivity within each ROI was quantified using per-sample pixel classifiers at a resolution of 2 μm/pixel. Intensity thresholds were manually adjusted for each slide, and positive pixel area was calculated as the percentage of total tissue area above the marker-specific threshold.

Statistical differences were analysed using a one-sample t-test on log2 fold changes. Conditions were compared to the vehicle control. Given the small sample size (n = 4) and the low number of glia-positive areas, #p < 0.1 was used in this analysis to identify trend-level effects. These results should therefore be considered exploratory and were not interpreted as statistically significant.

#### Mitochondrial stress test

MEHP effects on mitochondrial function were tested using ovarian cortex-derived primary ovarian cells in culture. Following dissociation of the ovarian cortex tissue, as previously described,^28^ primary ovarian cells were seeded at a density of 5 × 10^3^ cells/well in Agilent Seahorse 96-well XF Cell Culture Microplate (Agilent Technologies, Santa Clara, CA, USA) and cultured for six days in low glucose Dulbecco Modified Eagle Medium (Thermo Fisher Scientific, Waltham, MA, USA) supplemented with 10% foetal bovine serum and 1% penicillin streptomycin solution. MEHP was added to the media at concentrations of 1X, 10X, 100X and 1000X in 5–6 technical replicates per condition, to study concentration response. Primary ovarian cells from three biological replicates were plated.

Mitochondrial stress was assessed using the Agilent Seahorse XF Cell Mito Stress Test Kit (Agilent Technologies, Santa Clara, CA, USA), according to the manufacturer's protocol. The sensor cartridge was hydrated overnight in Seahorse XF Calibrant Solution at 37°C. On the day of the assay, cells were washed and incubated in Seahorse XF RPMI Assay Medium, supplemented with 10 mM glucose, 1 mM pyruvate, and 2 mM glutamine, for 45–60 min at 37°C to allow equilibration prior to the run. Measurements were carried out by Seahorse XFe96 Extracellular Flux Analyzer (Agilent Technologies, Santa Clara, CA, USA).

The oxygen consumption rate (OCR) was measured in real time before and after sequential injection of: oligomycin (1.5 μM), carbonyl cyanide-4-(trifluoromethoxy)phenylhydrazone (FCCP; 1.0 μM) and a combination of rotenone (0.5 μM) and antimycin A (0.5 μM). Three baseline measurements were recorded prior to the first injection, followed by three measurement cycles after each subsequent injection.

Basal respiration, maximal respiration, spare respiratory capacity, proton leak, and non-mitochondrial oxygen consumption were calculated based on manufacturer protocol.

Following the assay, cells were incubated with 5 μM Hoechst 33,342 (Thermo Fisher Scientific, Waltham, MA, USA) and imaged on a BioTek Cytation 5 imaging reader (Agilent Technologies, Santa Clara, CA, USA). Total cell nuclei number per well were counted using the integrated Gen5 software (version 3·17), and all OCR values were normalised to account for differences in cell density across wells.

#### Schwann cell differentiation and exposure

To validate MEHP effects on glial cells, a stem cell-based model was used. As peripheral tissue glia are most likely Schwann cells, a Schwann cell differentiation protocol was followed. Female human induced pluripotent stem cell (hiPSC) line CTRL14 was purchased from the Karolinska Institutet iPSC core facility. Cell lines generated by mRNA-mediated reprogramming at the facility are routinely tested for mycoplasma contamination, morphologically characterised, assessed for pluripotency marker expression by FACS, and authenticated (ethical licence 2012/208-31). CTRL14, previously published in,^37^^,^^38^ was transferred to the Department of Women's and Children's Health under a material transfer agreement. The cell line was provided for these studies by Assoc Prof. João Pedro Alves-Lopes. This cell line has been approved for research in accordance with Swedish legal requirements and the European regulation (EU) 2024/1938. No additional cell line validation was performed before the start of differentiation. Schwann cell differentiation was performed according to a previously published protocol with minor modifications.^39^ The initial differentiation step from primed stem cells to neural rosette cells was carried out using the STEMdiff™ Neural Crest Differentiation Kit (STEMCELL Technologies, Vancouver, Canada) according to the manufacturer's instructions. Differentiation of Schwann precursor cells and final maturation into Schwann cells were subsequently performed according to the protocol described previously.^39^

To mimic adult Schwann cell exposure, MEHP treatment was performed on day 6 of the final Schwann cell maturation stage using the same concentrations as in the ovarian explant culture experiments (1X and 1000X MEHP). Due to concerns regarding the stability of terminally differentiated Schwann cells in culture, a shorter exposure time of 48 h, instead of 6 days, was selected. Following exposure, cells were lysed in RLT buffer and RNA extraction was performed using the Qiagen RNeasy Micro Kit (Qiagen, Hilden, Germany) according to the manufacturer's instructions. cDNA synthesis and RT-qPCR were performed using the Bio-Rad iScript™ cDNA Synthesis Kit and SsoAdvanced Universal SYBR Green Supermix with a 384-well Bio-Rad RT-qPCR system (Bio-Rad, Hercules, California) according to the manufacturer's instructions. Primers used for Schwann cell differentiation markers are available in^39^ while primers used for MEHP-response analyses are listed in Supplementary Table S1.

### Statistics

Statistical analyses were performed using RStudio. Single-cell differential gene expression analysis was performed for each cell population compared to all others using Seurat's FindAllMarkers function with the Wilcoxon rank-sum test (unadjusted p < 0.05, log2FC > 2) to determine cluster-specific expression signatures. Metabolic activity scores were predicted from the count matrix using the scMetabolism package against the Kyoto Encyclopedia of Genes and Genomes (KEGG) dataset, employing the AUCell method. Associated statistical comparisons were performed using the Kruskal–Wallis test with Dunn's post hoc test, followed by Benjamini-Hochberg correction for multiple comparisons. Immunofluorescence data were first tested for normality using the Shapiro–Wilk test, followed by the Kruskal–Wallis test with Dunn's post hoc comparison. All conditions were compared to the vehicle control, with statistical significance defined as p < 0.05. qPCR and multiplex staining results were analysed using a one-sample t-test on log2 fold changes, with all conditions compared to the vehicle control. Statistical significance is indicated as follows: #p < 0.1, ∗p < 0.05, ∗∗p < 0.01, ∗∗∗p < 0.001.

### Role of funders

The funders had no role in the study design, data collection, analysis, interpretation, writing, or manuscript preparation.

## Results

### Human ovarian explants retain all expected cell types including glial cells

We prepared three separate single-cell libraries from ovarian tissue of one donor cultured for six days with two different MEHP concentrations or vehicle control. Integration of data from the three libraries resulted in the identification of 18 distinct cell clusters (Fig. 1A). As no comprehensive reference datasets for human ovarian cell types are currently available, we used well-established markers from the literature^24^^,^^28^^,^^29^ together with the HPA knowledge resource to annotate the clusters (Fig. 1B). This approach led to the identification of seven main cell populations: oocytes, granulosa cells, stromal cells, perivascular cells, endothelial cells, immune cells, and glial cells (Fig. 1C). Upregulated differentially expressed genes (DEGs) (p < 0.05, log_2_ fold change>2, Wilcoxon rank-sum test) were identified for each cell type (cell type markers) and used for identity validation through GO enrichment analysis. In total, 739 cell type markers were identified in oocytes compared to other cell types, 207 in granulosa cells, 38 in stromal cells, 110 in perivascular cells, 299 in endothelial cells, 314 in immune cells, and 290 in glial cells (Supplementary File S1A). GO enrichment analysis revealed characteristic cell type-specific ontologies (Fig. 1D, Supplementary File S1B). This validation was particularly important for recently described ovarian cell populations, such as glial cells, which displayed specific nervous system related terms including “axonogenesis” and “gliogenesis”. Similarly, the oocyte cluster was enriched for reproductive and meiotic terms, confirming the accuracy of the cell type annotation. Stromal cells showed enrichment for collagen-related terms, while immune cells were enriched for processes involving the regulation of leukocytes and T cells, along with other immune cell types (Fig. 1D, Supplementary File S1B). Endothelial cells were enriched for angiogenesis- and endothelium-related terms, perivascular cells for muscle-related terms, and granulosa cells for ‘regulation of hormone secretion’ and ‘positive regulation of cytokine production’ (Fig. 1D, Supplementary File S1B). Altogether, these analyses confirmed the presence of the main expected cell types in human ovarian cortical tissue after 6 days of culture. Notably, we also detected glial cells, a cell type only recently reported in human ovaries, thereby providing independent validation of their existence and demonstrating that they persist in ovarian explant cultures.

**Fig. 1:**
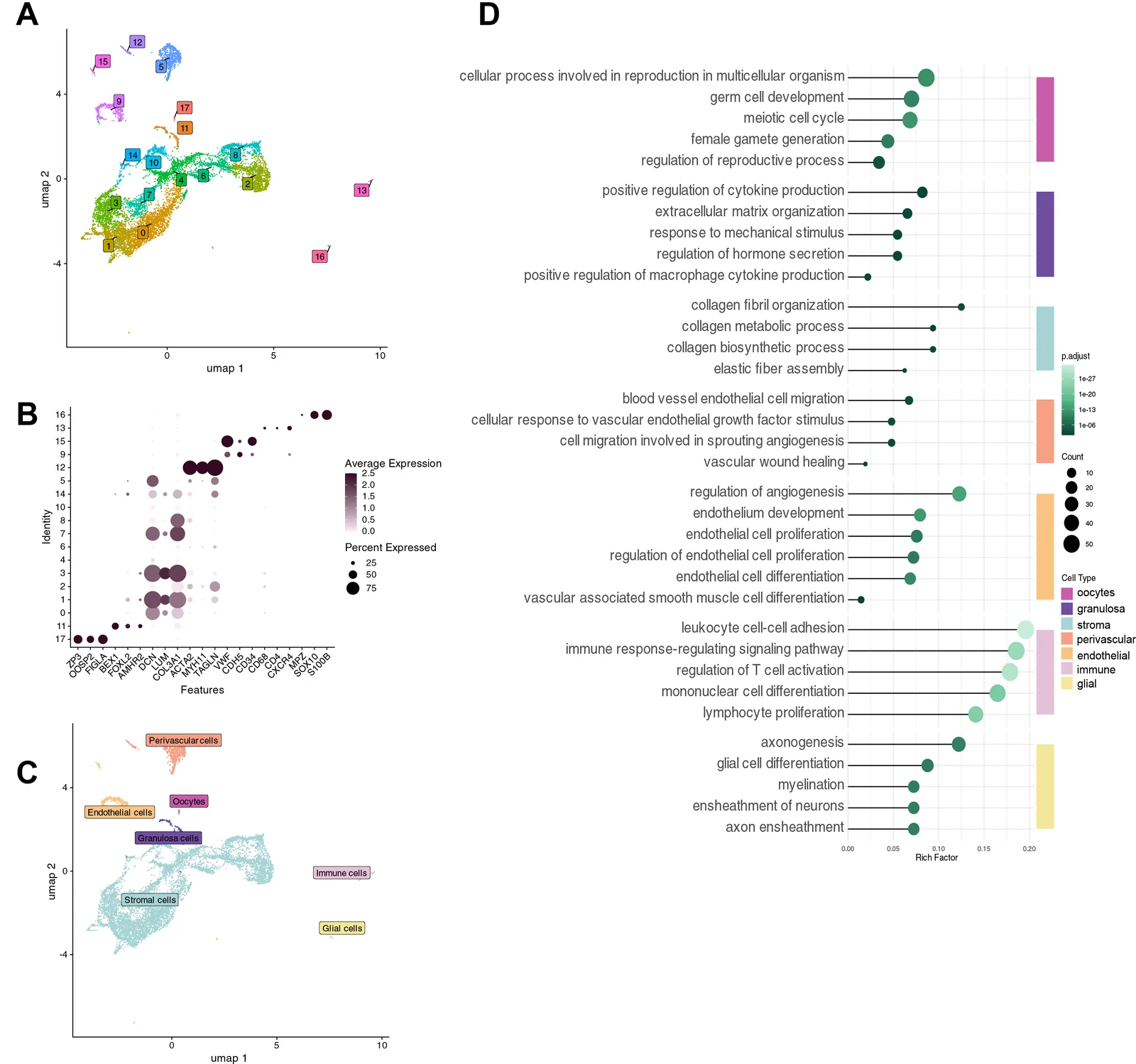
**Human ovarian explants retain all expected cell types including glial cells.** Human ovarian cortical tissue from a 23-year-old donor was exposed to 1X MEHP, 1000X MEHP or the vehicle DMSO for 6 days in culture. The explants were then collected for downstream single-cell RNA sequencing analysis. (A) UMAP plot of integrated datasets from ovarian tissue treated with 1X MEHP, 1000X MEHP, or DMSO control, showing the distribution of cell populations after quality control and filtering. “umap 1” and “umap 2” represent the two dimensions of the UMAP embedding. (B) Dot plot displaying the expression of canonical cell type markers across the 18 identified clusters. (C) UMAP plot showing the identified cell populations in explants, with each cluster colour-coded according to its assigned cell type. (D) GO enrichment analysis of upregulated genes (cell type markers) in each cell population, highlighting significant GO terms related to biological processes and supporting the accuracy of cell type annotations. Cell types are indicated by colour, from top to bottom: oocytes, granulosa, stroma, perivascular, endothelial, immune and glial cells.

### MEHP alters ovarian cell transcriptomes

We next assessed the effect of MEHP treatments on ovarian cell populations. All identified cell types were consistently present across all three conditions, DMSO, 1X MEHP and 1000X MEHP, with similar cell type proportions between control and exposure groups (Fig. 2A and B).

**Fig. 2:**
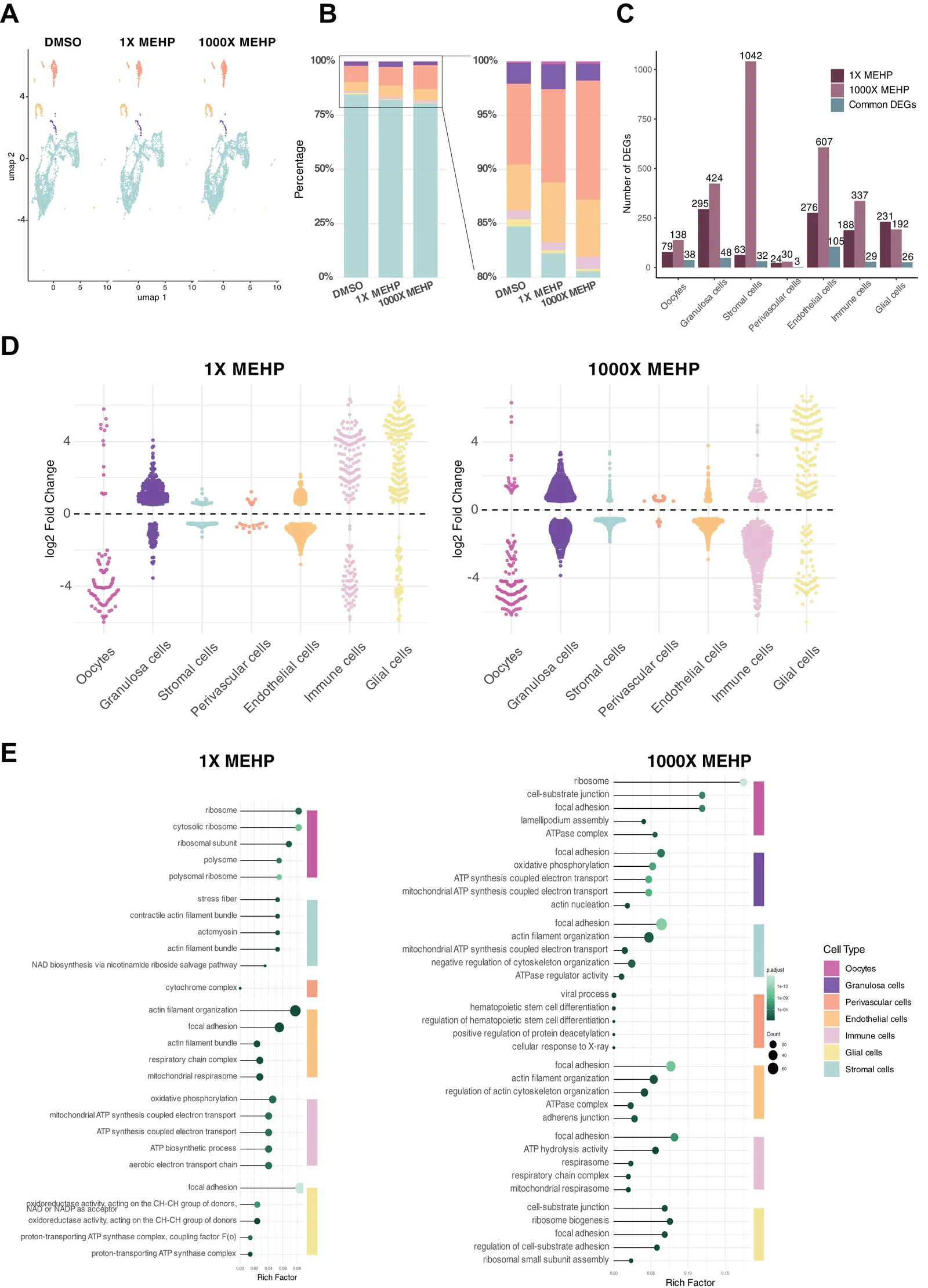
**MEHP alters ovarian cell transcriptomes.** Human ovarian cortical tissue explants derived from one 23-year-old donor were exposed to 1X MEHP, 1000X MEHP, or the vehicle DMSO for 6 days in culture, and the transcriptomic effects of the exposure were studied at single-cell resolution. (A) UMAP plots showing the distribution of cell populations across the three exposure conditions (DMSO, 1X MEHP, 1000X MEHP), with all treatments contributing to the main identified cell types. (B) Stacked bar plots displaying the proportions of each cell population within each exposure condition, illustrating the relative abundance of each cell type under DMSO, 1X MEHP, and 1000X MEHP. A zoomed-in view of the less abundant cell types is shown on the right. (C) Bar plots showing the number of DEGs (p < 0.05, |log_2_FC|>0.5, Wilcoxon rank-sum test) in the 1X and 1000X MEHP conditions, as well as their overlap within each cell type. In general, 1000X MEHP induced more DEGs than 1X MEHP, with limited overlap between conditions. Most DEGs were observed in stromal, endothelial, and granulosa cells. (D) Beeswarm plots showing the log2FC of individual DEGs per cell type for 1X MEHP and 1000X MEHP exposures. The fold changes were modest in cell types with many DEGs (stromal and endothelial cells), and more pronounced in rarer cell types with fewer DEGs (glial and immune cells). (E) Selected GO terms enriched among DEGs in the 1X MEHP (left) and 1000X MEHP (right) conditions, indicating the biological processes most affected by MEHP exposure within each cell type. Cell types are indicated by colour, from top to bottom: oocytes, granulosa, stroma, perivascular, endothelial, immune and glial cells. Commonly enriched terms included ribosome function, ATP synthesis, actin filament organisation, and cell adhesion. No GO terms were enriched for granulosa cells at the 1X MEHP concentration. The results are based on three separate libraries from one tissue donor with no replicates.

We then analysed differential gene expression between MEHP-treated explants and the DMSO control, identifying numerous DEGs (p < 0.05, |log_2_FC|>0.5, Wilcoxon rank-sum test) across all cell types. When visualising the numbers of DEGs unique to each condition (1X MEHP, 1000X MEHP) and those shared between them, it became evident that most DEGs were specific to each exposure level (Fig. 2C). Additionally, the 1000X MEHP exposure induced a greater number of DEGs than the 1X exposure in all cell types except glial cells, suggesting that these cells might be particularly responsive to low levels of exposure (Fig. 2C). Overall, the largest cell cluster, stromal cells, exhibited the highest number of DEGs at 1000X concentration, while perivascular cells exhibited the lowest number at both concentrations. Granulosa cells, which surround oocytes within follicles, exhibited more than three times as many DEGs as oocytes, suggesting granulosa cells are the most responsive cell type within follicles (Fig. 2C).

However, when considering fold changes as a measure of sensitivity, a different picture emerged (Fig. 2D). The largest fold changes were observed in the rarer cell types (immune cells, glial cells, and oocytes) whereas the lowest fold changes were seen in the larger cell populations such as stromal, perivascular and endothelial cells (Fig. 2D). At both exposure levels, glial cells, immune cells, and oocytes displayed the greatest fold changes in both up- and down-regulated genes in response to MEHP (Fig. 2D).

To investigate the processes affected by MEHP exposure, we carried out GO enrichment analyses for biological processes based on all the identified DEGs from the 1X and 1000X MEHP groups. These analyses revealed overlapping enrichment across all cell types in broad biological processes like transcription, translation, and ribosome-related terms (Supplementary File S2). Representative significant GO terms for each cell type are shown in Fig. 2E. The enriched GO terms for biological process indicated that cytoskeleton- and focal adhesion-related processes were affected in stromal, endothelial, and glial cells at both MEHP concentrations, as well as in oocytes and granulosa cells at the highest MEHP concentration. Similarly, OXPHOS-related terms were enriched across all cell types: in stromal, endothelial, and immune cells at both concentrations, in glial and perivascular cells at 1X MEHP, and in oocytes and granulosa cells at 1000X MEHP.

To further study MEHP impact on cellular energy metabolism, scMetabolism analysis was carried out to estimate metabolic pathway activities at the single-cell level. Consistent with the GO enrichment results, MEHP exposure led to significant alterations in OXPHOS pathways across most cell types. Specifically, metabolic pathway scores indicated increased OXPHOS activity in stromal, perivascular, endothelial, granulosa, and immune cells. However, no significant changes were detected in oocytes or glial cells (Supplementary Figure S1).

Altogether, MEHP exposure affected gene expression across all ovarian cell types, with the largest fold changes observed in the less common cell populations. Despite limited overlap in DEGs, the affected GO terms showed substantial similarities at both exposure levels, indicating that MEHP primarily influences pathways related to cell adhesion, cytoskeletal organisation, and mitochondrial function.

### Validation of MEHP effects on OXPHOS and cell adhesion proteins

Based on the single-cell RNA sequencing data, DEGs were selected for validation using an independent set of MEHP-exposed human ovarian tissues from six donors (Table 1). Targets were chosen based on differential expression between MEHP and DMSO conditions and their involvement in mitochondrial function or cell adhesion, as identified by GO enrichment analysis and literature. In addition, availability of high-reliability antibodies was taken into consideration. *VCL* was selected as a cell adhesion marker involved in actin cytoskeleton organisation and affected in oocytes, stromal cells, and glial cells (Supplementary Figure S2, Supplementary File S2). Several genes related to mitochondrial OXPHOS were significantly altered by MEHP across cell types, leading to the selection of the complex I components *MT-ND3* and *MT-ND4L*, and *EIF5A*, a translation factor linked to mitochondrial protein expression and OXPHOS regulation, for validation (Fig. 3A, Supplementary Figure S2, Supplementary File S2). For protein-level validation at the ovarian structure level, follicles and blood vessels were readily identified morphologically, whereas rarer cell types required co-staining. Therefore, MPZ and PTPRC were used as markers for glial and immune cells, respectively.

**Fig. 3:**
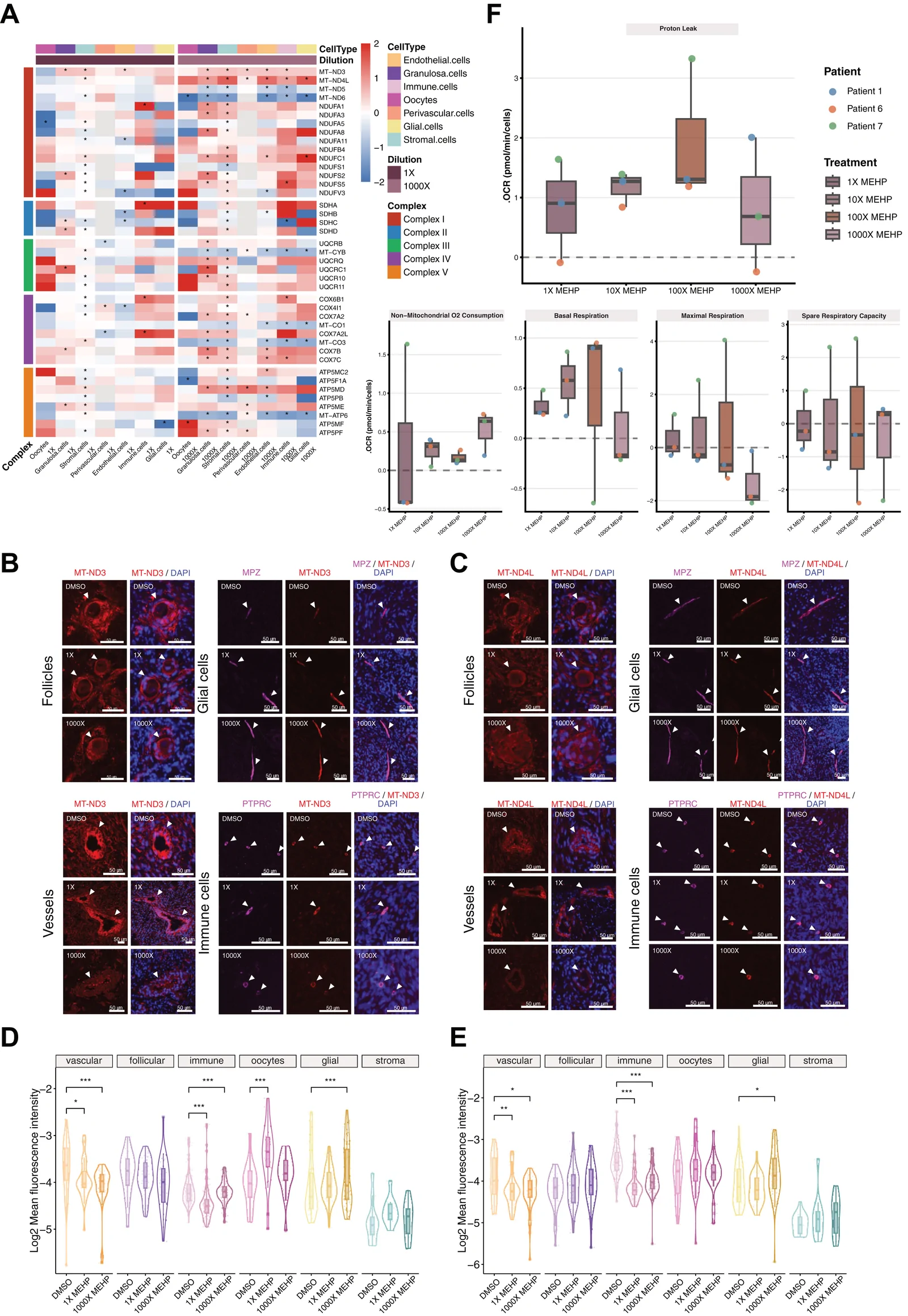
**MEHP exposure effects on mitochondrial genes and function.** Human ovarian cortical explants from three patients were exposed to 1X and 1000X MEHP for 6 days in culture. On the final day, tissue was collected for antibody-based detection of selected DEGs related to mitochondrial function. Tissue-dissociated primary cells from three patients were used to assess mitochondrial function following 6-day exposure. (A) Heatmap representation of oxidative phosphorylation complex genes in mitochondria, showing DEGs within each complex upon MEHP exposure 1X and 1000X with asterisks (cutoff p-value <0.05). (B, C) Expression of selected complex I genes MT–ND3 and MT–ND4L in MEHP-exposed tissue. Representative images from three patients are shown. Fluorescent signals are shown in follicles, vessels, immune, and glial cells, as indicated by arrows. For each structural category, the representative images per exposure condition are selected from the same patient (every row is a single patient), and the same patient order is maintained across all structural categories All images are presented with identical brightness and contrast adjustments. The scale bar represents 50 μm, and images were acquired at 20 × magnification. Figures were created using omero.figure. (D, E) Immunofluorescence signals were quantified using CellProfiler, either by manual segmentation of cell types or through co-staining with established markers: MPZ for glial cells and PTPRC for immune cells. Results are presented as violin plots with integrated boxplots. Each point in the jitter overlay represents one measurement: vascular cells from a distinct vessel, follicular cells from a single follicle, one oocyte, one group of immune or glial cells, or stromal cells from a single field of view. The total number of measurements per structure and protein is presented in Supplementary Table S2, and the contribution of individual patients to each structure is shown in Supplementary Figure S3. Statistical analysis indicated downregulation of MT–ND3 at both concentrations in vascular and immune cells, and upregulation in oocytes (1X) and glial cells (1000X). MT–ND4L was downregulated in vascular and immune cells at both concentrations and upregulated in glial cells at 1000X. Statistical analysis was performed using the Kruskal–Wallis test with Dunn's post hoc correction. Statistical significance was set at p < 0.05, with p < 0.05 indicated with ∗, p < 0.01 with ∗∗ and p < 0.001 with ∗∗∗. (F) Box plots showing mitochondrial respiratory parameters measured by Seahorse XF Cell Mito Stress Test Kit in primary ovarian cells following exposure to increasing concentrations of MEHP (1X, 10X, 100X, 1000X) for 6 days (n = 3). Parameters assessed include proton leak, non-mitochondrial oxygen consumption, basal respiration, maximal respiration and spare respiratory capacity. OCR values were normalised to cell number per well and expressed as pmol/min/cells. Data are presented as the difference to DMSO control, where the dash line represents zero, indicating no change in parameters. The coloured dots in each box correspond to individual patient samples.

Immunofluorescent staining of MEHP-exposed tissues validated altered protein expression of *MT-ND3* and *MT-ND4L*. Both proteins were down-regulated in vascular and immune cells, while upregulation was observed in glial cells (Fig. 3B–E). Additionally, MT-ND3 was upregulated in oocytes (Fig. 3B and D). Functional consequences were evaluated using a mitochondrial stress assay in primary ovarian stromal cells exposed to different MEHP concentrations (1X, 10X, 100X, and 1000X), with response calculated as difference to the DMSO control. Proton leak showed a concentration-dependent increase in median OCR values up to 100X MEHP concentration (Fig. 3F). Changes in other measured parameters, including non-mitochondrial oxygen consumption, basal respiration, and spare respiratory capacity, remained close to zero, except for maximal respiration, which displayed a concentration-dependent decline relative to DMSO. Considerable sample variability was noted across all treatment groups.

*VCL* was downregulated in oocytes and stromal cells according to the differential expression analysis (Supplementary Figure S2), with immunofluorescence validation showing reduced expression at 1X concentration in follicular cells and oocytes, and at 1000X concentration in vascular and follicular cells (Fig. 4A and C). Similarly, *EIF5A* was downregulated in endothelial cells in the single-cell RNA-sequencing data (Supplementary Figure S2), and immunofluorescence confirmed reduced protein levels in vascular cells, as well as in follicular cells and oocytes at the 1X concentration (Fig. 4B and D).

**Fig. 4:**
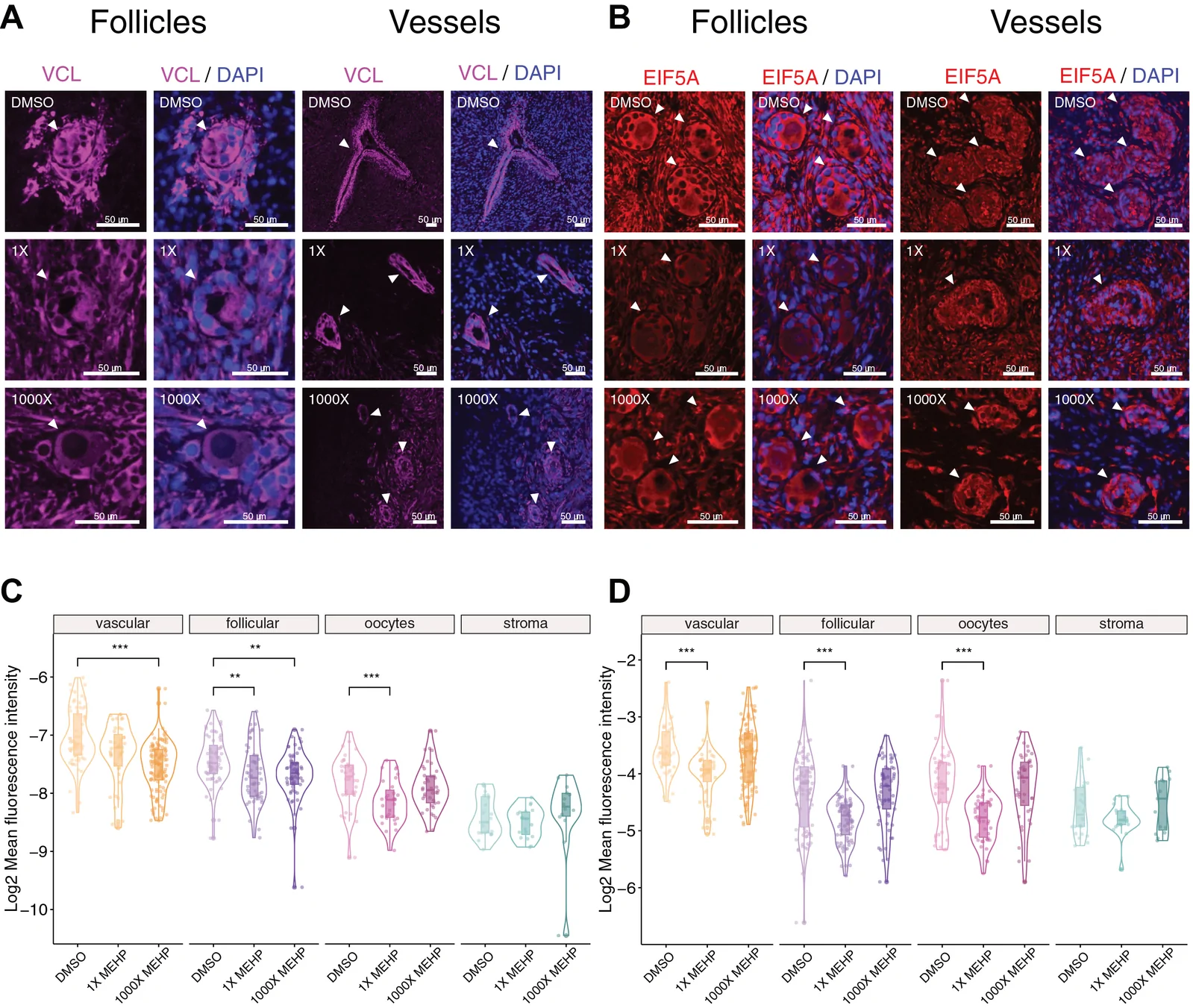
**MEHP exposure effects on VCL and EIF5A expression in ovarian tissue.** Human ovarian cortical tissue from six patients was exposed to 1X and 1000X MEHP for 6 days in culture. On the final day, tissue was collected for antibody-based detection of the selected DEGs. (A, B) Expression of VCL, related to cell junctions and actin cytoskeleton organisation, and EIF5A, a transcription factor controlling oxidative phosphorylation genes, is shown in MEHP-exposed cortical tissue from three representative patients. Staining was visualised in follicles and vessels, as indicated by arrows. For each structural category, the representative images per exposure condition are selected from the same patient (every row is a single patient), and the same patient order is maintained across all structural categories. All images are presented with consistent brightness and contrast adjustments. The scale bar represents 50 μm, and images were acquired at 20 × magnification. Figures were created using omero.figure. (C, D) Immunofluorescence signals were quantified using CellProfiler by manual segmentation. Results are presented as violin plots with integrated boxplots, overlaid with jitter plots where each point represents one measurement: vascular cells from a distinct vessel, follicular cells from a single follicle, one oocyte, and stromal cells from a single field of view. The total number of measurements per structure and protein is presented in Supplementary Table S2, and the contribution of individual patients to each structure is shown in Supplementary Figure S3. Statistical analysis indicated VCL downregulation at both concentrations in follicular cells, at 1000X in vascular cells, and at 1X in oocytes. EIF5A was downregulated at 1X in vascular cells, follicular cells, and oocytes. The Kruskal–Wallis test with Dunn's post hoc test was used. A p-value of p < 0.05 was considered significant, ∗∗ indicating p < 0.01 and ∗∗∗ indicating p < 0.001.

The total number of measurements per structure and protein is presented in Supplementary Table S2, and the contribution of individual patients to each structure is shown in Supplementary Figure S3. All immunostaining experiments included negative and secondary-only controls, which showed no detectable background signal, confirming the specificity of the staining (Supplementary Figure S4).

Overall, immunofluorescence staining validated the selected marker genes as protein-level targets of MEHP exposure in human ovarian tissue across multiple cell types. Consistent with the fold changes observed in the single-cell RNA-sequencing data, expression changes were more evident in the rarer cell types; glial cells, immune cells, vascular cells, and oocytes; compared to stromal cells. Despite smaller predicted changes in stromal cells based on scRNA-seq and the lack of protein-level validation, primary cell culture experiments supported functional effects of MEHP on mitochondrial proton leak and maximal respiration. These findings indicate that MEHP affects mitochondrial, cell adhesion and cytoskeletal pathways in these specialised cell populations, suggesting greater vulnerability to metabolic and structural disruption.

### MEHP reduces intercellular signalling in a concentration-dependent manner

To examine the effects of MEHP on cell–cell communication, we conducted CellChat analysis to assess changes in inferred interactions and communication strength across the treatment conditions (DMSO, 1X MEHP, and 1000X MEHP). Overall, MEHP exposure resulted in a concentration-dependent reduction in both the total number of interactions and interaction strength (Fig. 5A).

**Fig. 5:**
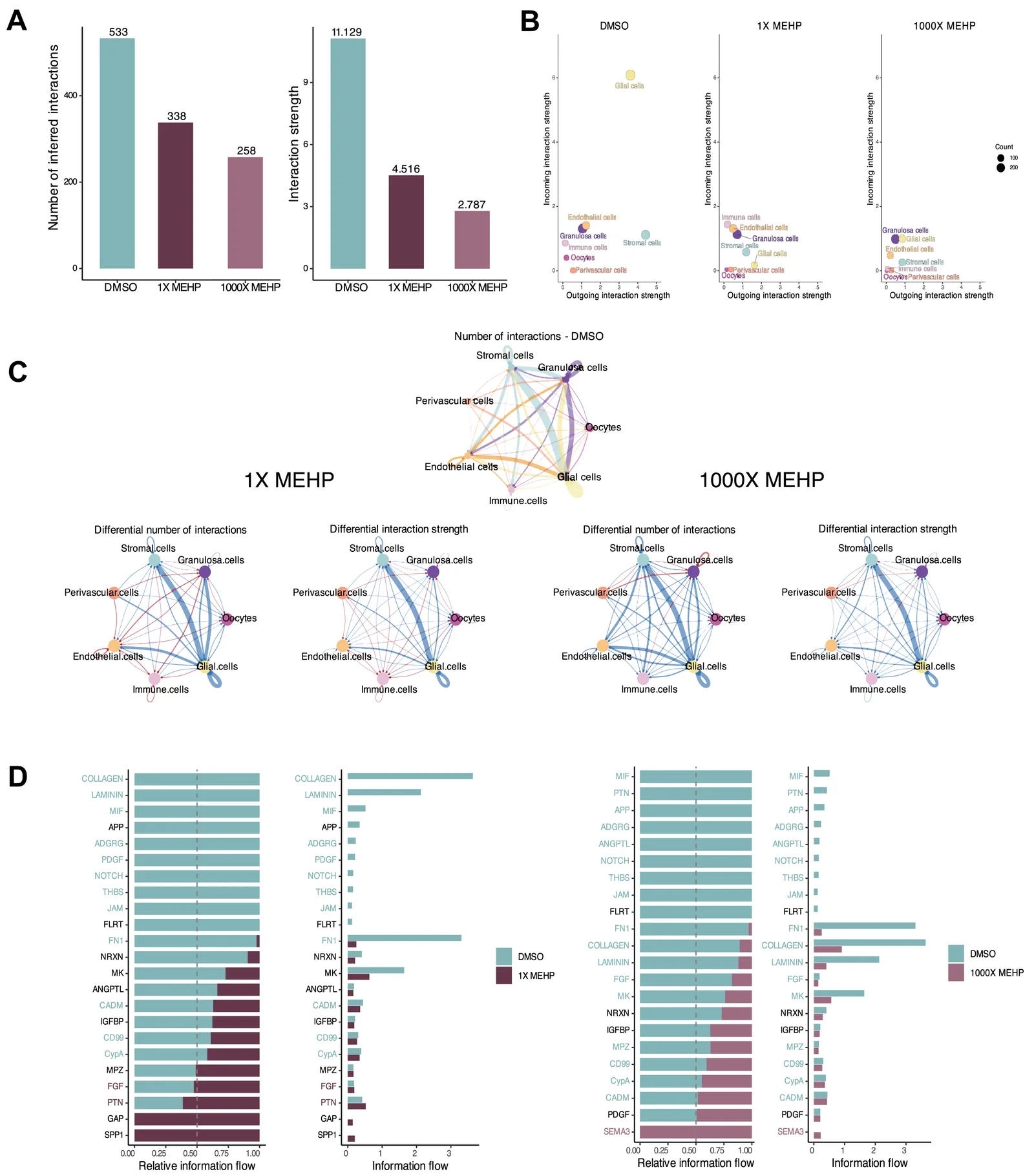
**MEHP reduces intercellular signalling in a concentration-dependent manner.** The scRNA-seq data from tissue explants of one 23-year-old donor, cultured for 6 days with 1X MEHP, 1000X MEHP, or DMSO vehicle, were analysed for predicted intercellular signalling. (A) Bar plots showing the number of inferred interactions (left) and the total interaction strength (right) for each treatment condition (DMSO, 1X MEHP, 1000X MEHP). Both the number and strength of interactions decrease in a concentration-dependent manner. (B) Scatter plots of incoming versus outgoing interaction strength for each cell type under different treatment conditions (DMSO, 1X MEHP, 1000X MEHP). A pronounced reduction in interaction strength was observed in glial cells following MEHP exposure. (C) Top: circle plot showing the baseline cell–cell communication networks in control explants (DMSO group). Arrow thickness represents the relative number or strength of interactions, and colours indicate the sending cell type. Arrow direction denotes outgoing or incoming signalling. Most interactions occurred between stromal, endothelial, granulosa, and glial cells. Bottom: circle plots showing the changes in cell–cell communication upon 1X and 1000X MEHP exposure compared with DMSO. Arrow thickness reflects the relative change in interaction number or strength, and arrow colour indicates whether signalling increased (red) or decreased (blue). The most prominent change under both MEHP conditions was the reduction in communication between stromal and glial cells. (D) Bar plots showing the significant signalling pathways altered in glial-stromal communication. The x-axis represents information flow for each condition (DMSO and MEHP) in relative and absolute terms. Bar colours indicate the contribution from each experimental group (DMSO vs. MEHP). Pathways are ranked by the difference in overall information flow between conditions. Pathway names significantly enriched under MEHP exposure are shown in purple, while pathways significantly enriched under DMSO (i.e., reduced under MEHP) are shown in blue. Pathways without significant differences between conditions are shown as black names. In both MEHP concentrations, signalling pathways related to extracellular matrix and cell adhesion were downregulated, whereas pathways related to neuronal stress responses were upregulated.

When communication activity was analysed by cell type, the most pronounced changes in both ingoing and outgoing interaction strengths were observed in glial cells, where the probability of incoming communication decreased by approximately sixfold and outgoing communication decreased by two-to threefold (Fig. 5B). We then analysed the strength and number of signalling events between cell types. As a first step, we examined signalling patterns in the control sample to establish a reference profile of communication activity in the explants. The strongest interactions were observed among glial cells and between glial cells and stromal, granulosa, and endothelial cells (Fig. 5C).

We next analysed the impact of MEHP exposure on this signalling landscape. Notably, MEHP exposure at both concentrations primarily reduced interactions among glial cells and between glial and stromal cells (Fig. 5C). Analysis of the signalling pathways most affected in glial–stromal communication revealed broad downregulation (Fig. 5D). Most downregulated pathways were shared between the two concentrations and were dominated by extracellular matrix and adhesion signalling, including collagen, laminin, thrombospondin (THBS), fibronectin (FN1), junctional adhesion molecule (JAM), adhesion G protein-coupled receptor G (ADGRG), cell adhesion molecule (CADM), and neuronal adhesion signalling via neurexin (NRXN). These changes may reflect the general disruption of cell adhesion by MEHP, consistent with the GO enrichment analyses (Fig. 2E).

Interestingly, the only pathways showing relative upregulation in response to MEHP included semaphorin 3 (SEMA3), significantly enriched at the 1000X MEHP concentration, and pleiotrophin (PTN) and fibroblast growth factor (FGF) at 1X MEHP. These pathways may indicate MEHP-induced stress-associated remodelling and communication program in ovarian glial cells. In addition, our recent transcriptomic analyses identifying *SEMA3* as one of the top predicted secreted signals from cortical ovarian follicles.^40^ This suggests a potential role for SEMA3 in ovarian glia-follicle signalling and warrants further study, both in terms of normal follicle physiology and as a target of chemical disruption.

To validate glia as a target of MEHP, an independent cell culture model was used. As peripheral glial cells are most likely Schwann cells, stem cell–derived Schwann cells model was employed for MEHP exposure studies (Supplementary Figure S5A–D). To mimic adult exposure, MEHP exposure was carried out using mature Schwann cells during the last 48 h of culture. All canonical Schwann cell maturation markers were upregulated, indicating that MEHP exposures were performed on cells that had successfully matured into a glial cell identity. Interestingly, *PLP* and *S100* were significantly further upregulated at 1X MEHP, with a similar trend observed for *GFAP*.

An additional set of markers representing high Log2FC MEHP-response genes in glia was included for validation, comprising mitochondrial (*TIMM10*, *SCL25A24*, *MRRF*), cell adhesion (*GYPC*, *CACNA2D1*, *FBLN2*, *THSD7A*) and cell communication-related genes (*CACNA2D1*). Comparison of qPCR results from iPSC-derived Schwann cells with scRNA-seq data from primary ovarian glial cells revealed directional concordance across all examined genes (Supplementary Figure S5E).

For example, *CACNA2D1*, a voltage-gated calcium channel subunit involved in calcium signalling, was upregulated by 1000X MEHP in both ovarian explant glial cells (0.0039, Wilcoxon rank-sum test) and the Schwann cell model (0.0617, Wilcoxon rank-sum test). *FBLN2*, coding for an extracellular matrix glycoprotein involved in cell adhesion, showed consistent downregulation across all three levels of evidence: transcript levels were significantly reduced in primary ovarian glial cells by scRNA-seq (p < 0.05, Wilcoxon rank-sum test), a concordant trend was observed by qPCR, and immunofluorescence confirmed reduced FBLN2 expression at 1X MEHP across four patients (p = 0.092, n = 4, one-sample t-test) (Supplementary Figure S5E and G). *VCL* and *EIF5A*, which showed differential expression in other ovarian cell types, displayed variable responses and did not show clear patterns in either system (Supplementary Figure S5E).

To investigate upstream mechanisms driving transcriptional changes in ovarian glial cells, we performed NicheNet ligand–receptor analysis (Supplementary Figure S5F) and examined differential expression of top-predicted ligands across ovarian cell types. DCN, encoding decorin, an ECM proteoglycan that sequesters TGFβ and modulates its bioavailability,^41^ was significantly upregulated in granulosa cells at both 1X (log2FC = 3.16, p = 0.0005, Wilcoxon rank-sum test) and 1000X MEHP (log2FC = 1.86, p = 0.039, Wilcoxon rank-sum test), suggesting reduced TGFβ signalling to glial cells across both doses. At 1000X, additional ligand expression changes were detected in stromal cells, where *SPON1*, *LAMB1*, *DLK1*, and *COL5A1* were significantly downregulated (all p < 10^−38^, Wilcoxon rank-sum test), and in endothelial cells, where *CD34* was downregulated. This pattern suggests that high-dose MEHP broadly disrupts ECM signalling and the paracrine niche supporting glial cells.

In addition, *AHR*, encoding the aryl hydrocarbon receptor, was significantly downregulated in glial cells at 1000X MEHP (log2FC = −2.52). MEHP effects on mouse follicles are affected by AHR,^42^ and AHR ligands also affect human follicles in tissue culture.^43^ Here, downregulation of the receptor in response to 1000X MEHP could indicate MEHP-induced receptor degradation and negative feedback regulation of the AHR signalling axis^44^ (Supplementary Figure S5F).

Together, these findings suggest a multifaceted mechanistic framework for MEHP-induced effects on ovarian glial cells. MEHP exposure disrupted cell adhesion and extracellular matrix components and caused a marked weakening of intercellular communication networks in the ovarian cortex. Glial cells appeared particularly sensitive to these changes. The loss of glial–stromal signalling likely reflects broadly impaired cellular connectivity and microenvironmental support, potentially involving extracellular matrix-regulated growth factors such as TGF-β, as well as xenobiotic sensors including AHR.

## Discussion

To enhance chemical regulation, testing and risk assessment, increasing our knowledge of phthalate toxicity is crucial. The phthalate metabolite MEHP is a known male reproductive toxicant that interferes with testicular development by lowering androgen levels.^45^ However, its effects on human ovarian function remain inadequately characterised, despite detection at relatively high levels in women.^2^^,^^21^ This study elucidates the impact of MEHP on adult human ovarian tissue using single-cell RNA-sequencing and a translational human explant model. Prior to this investigation, studies of phthalate effects on human ovaries at single-cell resolution were lacking.

The MEHP concentrations used in this study reflect human-relevant exposure levels: the 1X concentration corresponds to the range observed in serum and follicular fluid of women of reproductive age (1.73 nM–75.45 nM)^8^^,^^46^, ^47^, ^48^, ^49^ and the 1000X concentration reflects levels measured in blood transfusion patients.^50^ In our previous study, MEHP exposure reduced follicular growth at the 1X concentration and increased follicle death at 1000X.^22^ Bulk RNA-sequencing of 2D ovarian granulosa and stromal cell cultures suggested that these effects were linked to gene expression changes related to cytoskeletal organisation and adherens junctions.^22^ In the current study, single-cell analysis of 3D ovarian explants confirmed these hypotheses and further revealed significant alterations in key mechanosensory components (*VCL*) and OXPHOS pathways across distinct ovarian cell populations at both concentrations. Interestingly, changes in energy metabolism were also observed in ovarian explants exposed to persistent organic pollutants in our previous studies, suggesting that this could represent a general response to environmental chemical exposure in the adult ovary.^22^^,^^51^

We found that proton leak was elevated in a concentration-dependent manner in primary ovarian cells exposed to MEHP. Proton leak reflects the portion of mitochondrial oxygen consumption that is uncoupled from ATP synthesis, arising from protons entering the mitochondrial matrix through pathways other than ATP synthase, causing dissipation of mitochondrial membrane potential.^52^ In this context, the elevated proton leak following exposure to MEHP suggests a perturbation of the inner mitochondrial membrane integrity in primary ovarian cells, potentially reflecting an early stress response.^53^ The primary ovarian cell cultures mainly consist of stromal cells, which were predicted to be among the least sensitive cell types to MEHP in our study. This highlights the need to assess MEHP effects on mitochondrial respiration in additional cell types, including granulosa and glial cells. For example, phthalates have been shown to alter oxygen consumption in primary mouse granulosa cells.^54^

Reproductive effects of DEHP have been studied at single-cell resolution in both testes and ovaries of mice.^55^, ^56^, ^57^ In testes, DEHP downregulated genes related to cell adhesion and cytoskeleton maintenance in Sertoli and Leydig cells, a pattern we also observed across several ovarian cell types, suggesting shared modes of action across sexes.^57^ Two single-cell studies performed on foetal mouse ovaries reported that DEHP impaired follicle assembly, increased DNA damage in pregranulosa and germ cells, and induced apoptosis.^55^^,^^56^ Reduced follicular growth and increased apoptosis were also observed in our earlier study using adult human tissue,^22^ highlighting that phthalates can disrupt ovarian development and function at multiple life stages. One foetal study further reported enrichment of GO terms related to OXPHOS, mitochondrial genome maintenance, and adherens junctions in germ cells, indicating that comparable biological processes are dysregulated in both foetal and adult ovarian tissue.^56^ This shows that the ovary remains sensitive to chemical exposures throughout life.

A recent study using single-cell sequencing of cultured mouse antral follicles exposed to a phthalate mixture found disruptions in cell-cycle, ribosomal, and endoplasmic reticulum-related processes in granulosa cells.^58^ However, this study did not detect oocytes or glial cells, and other cell types, including immune, endothelial, epithelial, and mesenchymal cells, were not further analysed. This is in line with our review of the literature; previous studies have rarely examined phthalate effects in ovaries beyond the follicles.^25^ A comprehensive understanding of chemical impacts on ovarian function requires insights into the roles these additional cell types play in fertility and the adverse outcomes resulting from their disruption.

Single-cell RNA-sequencing offers a unique opportunity to dissect chemical responses at cell-type resolution and detect subtle molecular changes that bulk analyses may overlook. In pharmacology, single-cell enrichment scores have already been used to predict heterogeneous drug responses.^59^ Applying similar approaches to environmental toxicants could pinpoint sensitive cell types and guide the development of more targeted testing strategies. However, a key challenge in single-cell transcriptomics within toxicology is the potential alteration of cell-type proportions and marker expression following exposure.^60^ To ensure accurate cluster identification, we validated cluster identification through GO enrichment, ensuring accuracy and reliability in our findings. An additional challenge arises from differences in cell cluster size, as larger clusters inherently have greater statistical power to detect changes in gene expression, which was also evident in our study. Despite the high number of DEGs identified in stromal cells, fold changes remained modest, and selected targets could not be robustly validated.

The single-cell approach identified glial cells, a rare and previously unexplored cell type in ovarian toxicity research. The ovary is innervated by nerve fibres that enter through the hilum, and hypothalamic neuronal growth factors have been implicated in conditions such as polyendocrine metabolic ovarian syndrome (PMOS).^61^ However, single-cell level detection of neuronal cells in human ovaries has so far been lacking,^23^ and the potential role of glial cells in the regulation of ovarian function remains unknown, particularly at the local, intra-ovarian level. A 2025 paper reported glial cells associated with sympathetic neurons spanning the medulla and cortex in human and mouse ovaries.^24^ Based on DEG fold changes and cell–cell communication analysis, our data suggest that glial cells represent the ovarian cell population most vulnerable to MEHP exposure. Neuronal cells have not previously been considered targets of endocrine disruptors in ovaries, although neurodevelopment is widely recognised as particularly sensitive to endocrine disrupting chemicals.^62^ The identification of ovarian glial cells and their sensitivity to chemical exposures may help explain the associations observed between phthalate exposure, ovarian sensitivity index, and PMOS.^8^^,^^63^, ^64^, ^65^

We selected *VCL* for validation due to its role as an actin-binding protein enriched in focal adhesions and adherens junctions.^66^ Our earlier data suggested that MEHP disrupts cytoskeletal and junctional integrity in granulosa cells, potentially impairing oocyte–granulosa communication.^22^ In the present study, *VCL* downregulation was detected in oocytes and stromal cells at the RNA level and confirmed at the protein level in vascular, follicular and oocyte compartments. These results validate our previous findings and extend them to additional ovarian cell types. Cytoskeletal disruption has also been observed in uterine cells exposed to another DEHP metabolite, MEHHP.^22^^,^^67^ Studies in uterine spheroids and isolated membranes have reported altered membrane fluidity,^68^ which may underlie impaired adhesion, as cytoskeletal–membrane interactions are essential for adhesion processes.^69^ Given that VCL even acts as a mechanotransducer, our results suggest that MEHP may interfere with ovarian mechanosensing, a process increasingly recognised as fundamental to folliculogenesis, thereby unveiling a potential new mode of action.^70^

The observed cytoskeletal dysregulation may also contribute to mitochondrial effects, as actin filaments are critical for mitochondrial positioning and bioenergetics.^71^ Cytoskeletal instability has likewise been linked to reduced mitochondrial function in aged mouse oocytes.^72^ GO enrichment analysis revealed alterations not only in OXPHOS and ATP production but also in ribosome-related events, particularly in oocytes. Interestingly, *EIF5A*, one of the validated targets, is essential for translation elongation, and its downregulation can cause ribosomal stalling.^73^ Ribosomal stalling, which also increases with age, may trigger compensatory ribosome synthesis.^74^ *EIF5A* was previously identified as the only common DEG among four human ovarian 2D cell cultures exposed to 1000X MEHP.^22^ It facilitates translation of mitochondrial proteins, including those involved in OXPHOS, suggesting it could act as an upstream regulator of the observed mitochondrial effects.^75^ Post-translational dysregulation of *EIF5A* has been linked to mitochondrial dysfunction and apoptosis, and cytoskeletal instability.^76^ Taken together, MEHP exposure may accelerate molecular signatures of ovarian ageing and exacerbate age-related decline in oocyte quality by disrupting both cytoskeletal integrity and mitochondrial function.^66^ This mechanistic disruption aligns with epidemiological associations of phthalate exposure with infertility, miscarriage and earlier reproductive ageing in women.^19^

Differential expression analysis revealed changes across all OXPHOS complexes, including assembly factors and ATP/ADP transporters. We validated two mitochondrial complex I genes, *MT-ND3* and *MT-ND4L*, which were upregulated in most cell types at the RNA level. In mice, downregulation of these genes in oocytes has been associated with ovarian ageing.^75^ Although these transcripts were not differentially expressed in oocytes in our data, immunofluorescence quantification showed *MT-ND3* protein upregulation at the 1X concentration. Because oocytes rely on granulosa cells for metabolic substrates, transcriptomic changes may not fully reflect their energy status.^75^ Notably, MT-ND3 and MT-NDL4L proteins were upregulated in glial cells, but downregulated in vascular and immune cells despite RNA upregulation. Differences between RNA and protein levels may arise from post-translational regulation, compensatory mechanisms, or heterogeneity in responses among follicles and other structures.^40^ Nevertheless, both RNA and protein results identify mitochondrial complex I as a consistent MEHP target, and metabolic prediction analysis confirmed upregulation of OXPHOS in most ovarian cell types. Such OXPHOS impacts could also disrupt calcium homoeostasis, as previously reported in oocytes exposed to DEHP.^77^^,^^78^

Recent human studies have reported associations between DEHP metabolite levels in follicular fluid and follicular hormone sensitivity, accompanied by molecular alterations within the follicles, including downregulation of cholesterol biosynthesis, disrupted progesterone metabolism, reduced ATP synthesis, and increased inflammation.^64^ Our current data did not indicate effects on cholesterol or steroid metabolism, likely because these processes are most active in gonadotropin-dependent antral follicles, not present in the ovarian cortex. However, the consistent targeting of OXPHOS across multiple ovarian cell types, in 2D cultures, tissue explants, and patient-derived follicular fluid, suggests not only shared mechanisms but also parallel pathways that together contribute to adverse outcomes. MEHP thus appears to act simultaneously as an endocrine disruptor (impaired steroidogenesis), classical toxicant (mitochondrial dysfunction), and even a “mechanodisruptor” (cell adhesion effects). Importantly, these effects occur at human-relevant exposure levels in short exposure settings, highlighting the urgency of strengthened chemical regulation to safeguard fertility.

Future work should aim to further clarify MEHP's mechanism of action using structural and functional endpoints related to cell adhesion, membranes, cytoskeleton, mechanosensing, energy metabolism, and cell signalling. Approaches such as cell painting or electron microscopy could reveal structural alterations. Mapping the localisation of neuronal cells and their secreted growth factors could further clarify their functions and consequences of their disruption. Investigating earlier time points with single-cell RNA sequencing could distinguish primary from compensatory responses and identify the most critical molecular targets and cell types. Rescue experiments using antioxidants or ECM modifying agents could test whether these effects are reversible. Finally, testing a broader repertoire of chemicals will help determine how general the identified cellular responses are across chemical categories. Given that the currently available human ovarian cell models do not include all cell types, particularly glial cells, future isolation and immortalisation of the rare but sensitive cell populations are warranted. Such models will enable both toxicological testing and exploration of local neuro–ovarian regulation.

This study has many limitations. Due to restricted access to human ovarian tissue, single-cell profiling was performed using material from one donor. We selected the youngest available donor to maximise physiological relevance for reproductive age females. All gender-affirming surgery patients had received androgen therapy prior to surgery, which could affect the results. However, our previous studies have not found major differences in ovarian cortical cell populations between c-section and gender-affirming care patients, or in vitro responses to stressors.^22^^,^^28^ In addition, independent cell models as well as c-section tissue were used to validate findings. Only one DEHP metabolite was tested, although DEHP is extensively metabolised into several monoesters, including oxidised forms. Mixture toxicity remains a critical challenge: combined effects of chemicals present in consumer products often differ from those of individual components.^79^^,^^80^ Studying single metabolites like MEHP, however, is essential for dissection mechanisms underlying mixture effects. Our earlier work with other phthalate metabolites and mixtures suggests that mitochondria and cell adhesion are recurrent toxicity targets.^67^^,^^68^ The explant culture system, while highly translational, is sensitive to inter-individual variation; we mitigated this by validating findings in tissues from a large number of individuals from different patient groups. While variability existed between individual samples in the number of ovarian structures, such as follicular and vascular regions, the sequencing results were positively validated across different individuals at the protein level. These findings indicate that MEHP induces robust changes in ovarian tissue that are detectable at both the RNA and protein levels across multiple samples. Finally, due to extensive environmental contamination, all humans are exposed to phthalates. We did not measure phthalate exposure levels of the tissue donors in our study. As phthalates are excreted within 24 h of exposure in humans, it is unlikely that patient-derived tissue phthalates would have affected the results in our 6-day in vitro exposure setting.

Strengths of this study include using human primary tissue as a model and validation of the single-cell findings using independent tissue sets from different donor types (gender-affirming and c-section patients) and separate stem cell-derived Schwann cell culture system. Both patient groups provided morphologically and functionally normal ovaries from reproductive-age individuals for the explants, a model representing the closest possible approximation of healthy human ovaries. Fresh tissues were processed directly after surgery for cultures, avoiding potential confounding effects of cryopreservation or temperature fluctuations during transport. Access to patient records confirmed the absence of diseases affecting the ovaries. We also developed a co-staining protocol enabling protein-level validation with cell- and structure-level precision. An additional strength of the study is the use of epidemiologically relevant exposure levels, which also presents a challenge. Effects of common environmental contaminants are not acutely toxic but rather develop over time. Capturing such effects in short-term in vitro cultures is a key challenge in toxicological sciences and necessitates identification and validation of subtle biological alterations.

Despite these challenges, we assessed MEHP effects at cell type resolution at both RNA and protein levels in reproductive-age human ovarian cortex, providing a uniquely translational toxicological dataset. In conclusion, MEHP exposure dysregulated processes related to mechanosensing, cell adhesion, actin cytoskeleton, OXPHOS and intercellular communication. Consistent with our previous studies, these effects extend beyond classical endocrine disruption to involve core cellular architecture and metabolism, relevant across all cell types. Notably, they occur at exposure levels found in women of reproductive age.^22^ We propose that MEHP acts through multiple interconnected mechanisms, highlighting the need to define the most critical effects and adverse outcomes for improved chemical testing and risk assessment. Validation of selected DEGs at the protein level further highlighted the sensitivity of underexplored ovarian cell types like glial cells. Current adverse outcome pathways describing female reproductive toxicity focus on follicles and steroidogenesis, overlooking these cell populations and mechanisms (https://aopwiki.org/). Our findings therefore broaden understanding of how phthalates disrupt ovarian function and emphasise the importance of viewing the ovary as a complex multicellular organ to better predict and prevent reproductive toxicity.

## Contributors

**Cecilia Lindskog, Kiriaki Papaikonomou, Catarina Arnelo, Karin Pettersson:** Resources. **Pauliina Damdimopoulou:** Writing—review & editing, supervision, funding acquisition, conceptualisation. **Eleftheria-Maria Panagiotou:** Writing—original draft, visualisation, investigation, formal analysis, data curation. **Loren Méar, Ilmatar Rooda, Alesandro Haxhiu, Karolina Wasilewska, Tianyi Li:** Visualisation, investigation, formal analysis, data curation. **Elisabeth Moussaud-Lamodière:** Formal analysis. **Eleftheria-Maria Panagiotou, Loren Méar, Ilmatar Rooda, and Pauliina Damdimopoulou** accessed and verified the underlying data. All authors read and approved the final version of the manuscript.

## Data sharing statement

The processed single-cell RNA sequencing data have been deposited in the ArrayExpress database under accession number E-MTAB-15922. The corresponding raw sequencing data constitute sensitive personal data and have been deposited in the Swedish National Data Service (SND) DORIS repository under the DOI https://doi.org/10.48723/3vtm-jm80. Access to the raw data may be granted upon request following review by Karolinska Institutet and subject to applicable ethical, legal, and data protection requirements.

The code used to reproduce the analyses and figures presented in this study is available on GitHub at https://github.com/DamdimopoulouLab/scMEHP.

## Declaration of interests

The authors report no financial or personal relationships that could inappropriately bias the work.
