# Supplementary material for: Single-cell study of human ovarian response to phthalate exposure reveals glial cell susceptibility and disruption of adhesion and mitochondrial pathways
Source: eBioMedicine. 2026 Oct 6;132:106487. doi: 10.1016/j.ebiom.2026.106487 (PMC13688040; doi:10.1016/j.ebiom.2026.106487)
Supplement: Supplementary file 1 [file mmc3.pdf]

# Single-cell study of human ovarian response to phthalate exposure reveals glial cell susceptibility and disruption of adhesion and mitochondrial pathways

Eleftheria Maria Panagiotou, Loren Méar, Ilmatar Rooda, Alesandro Haxhiu, Elisabeth Moussaud-Lamodière, Karolina Wasilewska, Tianyi Li, Catarina Arnelo, Karin Pettersson, Kiriaki Papaikonomou, Cecilia Lindskog, Pauliina Damdimopoulou

## Supplementary Tables and Figures

**Page 2** **Supplementary Table 1.** Information on the antibodies used including catalogue numbers, species they were raised and concentrations used.

**Page 3** **Supplementary Table 2.** Number of datapoints used for the quantification of the immunofluorescence images.

**Page 4** **Supplementary Figure 1**. Predicted OXPHOS pathway activity in human ovarian cell types after 6 days of explant culture.

**Page 5** **Supplementary Figure 2**. Expression of *VCL, EIF5A, MT-ND3* and *MT-ND4L* by cell type in MEHP-treated human ovarian explants.

**Page 6** **Supplementary Figure 3.** Number of datapoints used for the quantification of the immunofluorescence images per patient.

**Page 7** **Supplementary Figure 4.** Negative controls of immunofluorescence staining on human ovarian cortical tissue exposed to DMSO for 6 days.

**Page 8-9** **Supplementary Figure 5.** Glial cell differentiation and exposure to MEHP

**Supplementary Table 1.** Information on the antibodies used including catalogue numbers, species they were raised and concentrations used, as well as primers used for qPCR analysis.

| Antibody | Catalogue number | Supplier | Species | Concentrations | RRID |
|---|---|---|---|---|---|
| Anti-MTND3 | HPA063296 | Sigma-Aldrich | rabbit | 1:50 | AB_2684978 |
| Anti-MTND4L | HPA075648 | Sigma-Aldrich | rabbit | 1:100 | AB_2686760 |
| Anti-VCL | HPA002131 | Sigma-Aldrich | rabbit | 1:250 | AB_1080570 |
| Anti-EIF5A | HPA074052 | Sigma-Aldrich | rabbit | 1:100 | NA |
| Anti-PTPRC | HPA000440 | Sigma-Aldrich | rabbit | 1:200 | AB_611377 |
| Anti-MPZ | HPA068925 | Sigma-Aldrich | rabbit | 1:2000 | AB_2732123 |
| Anti-CD34 | M7165 | Agilent | mouse | 1:900 | AB_2063006 |
| Anti-FBLN2 | HPA001934 | Sigma-Aldrich | rabbit | 1:250 | AB_1078827 |
| Anti-rabbit 555 | A31572 | Thermo Fisher | donkey | 1:200 | NA |
| Anti-rabbit 647 | A31573 | Thermo Fisher | donkey | 1:200 | NA |
| Rabbit IgG | ab109489 | Abcam | donkey | 1:50 | NA |

| GENE | FORWARD 5' - 3' | REVERSE 5' - 3' |
|---|---|---|
| VCL | TGAGCAAGCACAGCGGTGGATT | TCGGTCACACTTGGCGAGAAGA |
| EIF5A | GAGCAGAAGTACGACTGTGGAG | CAGGTTCAGAGGATCACTGCTG |
| MT-ND3 | ACTTTCACCGCTACACGACCG | CAGTGGGCTCTAGAGGGGGT |
| MT-ND4L | ACTTTCACCGCTACACGACCG | CAGTGGGCTCTAGAGGGGGT |
| THSD7A | GTCCCCTGTGCCACGTATGG | ACGCAGTACACCTCTCGGGT |
| GYPC | TGGCCTCTCAGCCTCGGAAGTC | GAATTCAGGCACAGGGTGAGGG |
| CACNA2D1 | CCACGTTTTACACTGTGCCCC | TCCTGAGATTTGGGGTTCTTTGGC |
| FBLN2 | GTGCCCCGCGGGTCTTAC | AATGCAGTTCTCCAGCGGCG |
| TIMM10 | TCCGTGGGCATTGTGATCCG | GGCAGGCACTGGTCATTCTGT |
| SLC25A24 | CCAGGACGCCGAGGAGAAAAT | AGTGTCTGGAGAGACTGGACAAT |

| | | |
|---|---|---|
| MRRF | TGGGCGATAGCTGAT GAGACG | ACTCCGAGTCTTGGTTCCAG C |

**Supplementary Table 2.** Number of datapoints used for the quantification of the immunofluorescence images.

VCL

| | endothelial | granulosa | oocytes | stroma |
|---|---|---|---|---|
| DMSO | 67 | 60 | 36 | 24 |
| 1X MEHP | 43 | 56 | 31 | 19 |
| 1000X MEHP | 120 | 60 | 40 | 14 |

EIF5A

| | endothelial | granulosa | oocytes | stroma |
|---|---|---|---|---|
| DMSO | 48 | 87 | 49 | 26 |
| 1X MEHP | 39 | 80 | 49 | 22 |
| 1000X MEHP | 104 | 61 | 43 | 15 |

MT-ND4L

| | endothelial | granulosa | oocytes | stroma | immune | glial |
|---|---|---|---|---|---|---|
| DMSO | 43 | 45 | 30 | 18 | 158 | 54 |
| 1X MEHP | 36 | 65 | 35 | 12 | 79 | 34 |
| 1000X MEHP | 57 | 41 | 23 | 16 | 102 | 52 |

MT-ND3

| | endothelial | granulosa | oocytes | stroma | immune | glial |
|---|---|---|---|---|---|---|
| DMSO | 44 | 18 | 13 | 10 | 225 | 63 |
| 1X MEHP | 31 | 55 | 31 | 10 | 138 | 40 |
| 1000X MEHP | 51 | 34 | 19 | 14 | 106 | 89 |

## Supplementary Figure 1

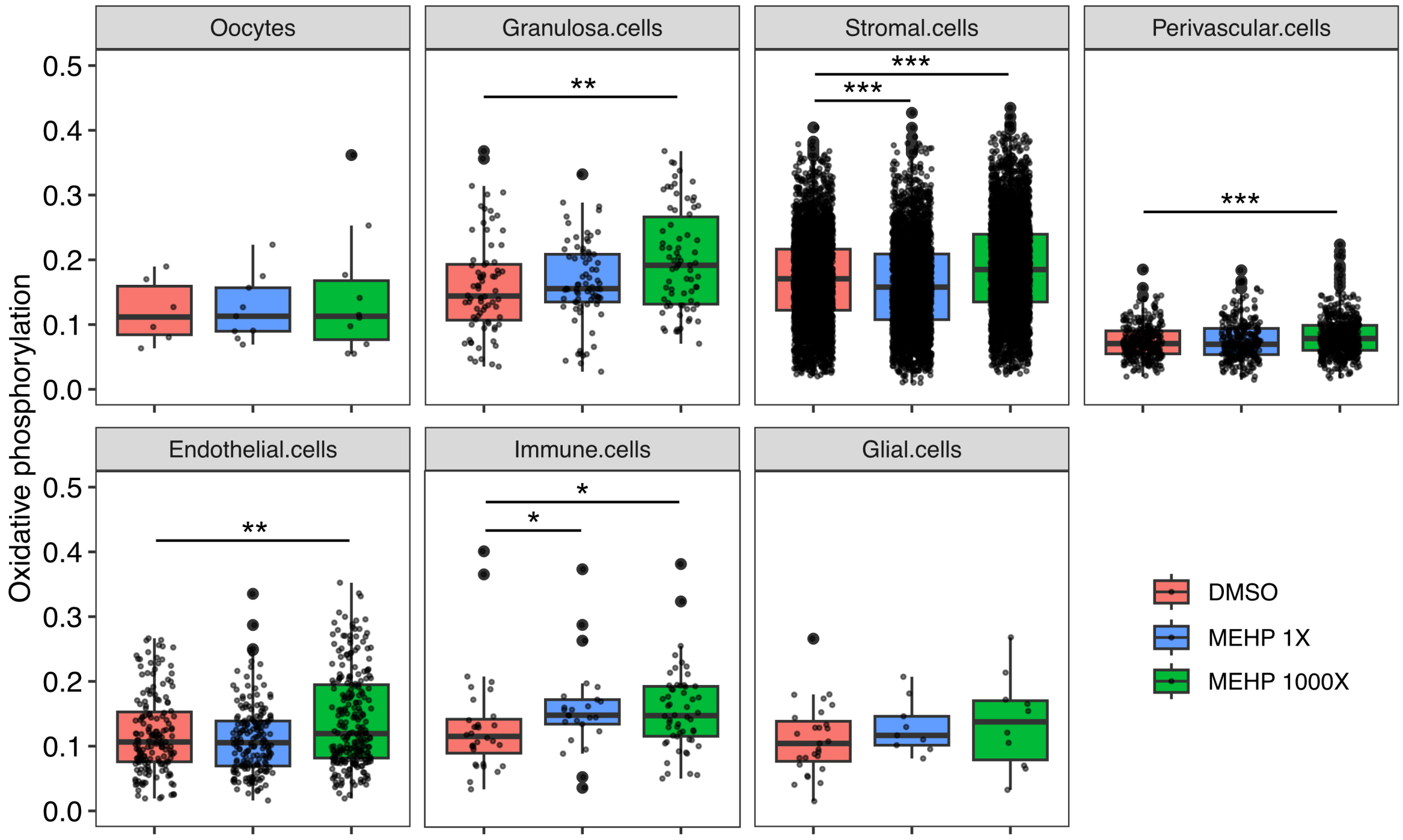


**Supplementary Figure 1. Predicted OXPHOS pathway activity in human ovarian cell types after 6 days of explant culture.**

Box plots show OXPHOS metabolic scores for each cell type under DMSO, 1× MEHP, and 1000× MEHP conditions. Metabolic scores were predicted using the scMetabolism package on the count matrix against the Kyoto Encyclopedia of Genes and Genomes (KEGG) dataset using the AUCell method. The results are presented as boxplots where boxes representing the interquartile range (IQR, 75$^{th}$ percentile – 25$^{th}$ percentile), the middle line shows the median, whiskers mark the non-outlier range, and outliers as large black dots. Individual cells were shown as small light-grey dots. Statistical comparisons were performed using Kruskal–Wallis test with Dunn's post hoc test, followed by Benjamini–Hochberg correction for multiple comparisons, and marked as * $p<0.05$; ** $p<0.01$ and ***$p<0.001$. Significant increases in predicted OXPHOS activity were observed in all cell types except oocytes and glial cells.

## Supplementary Figure 2

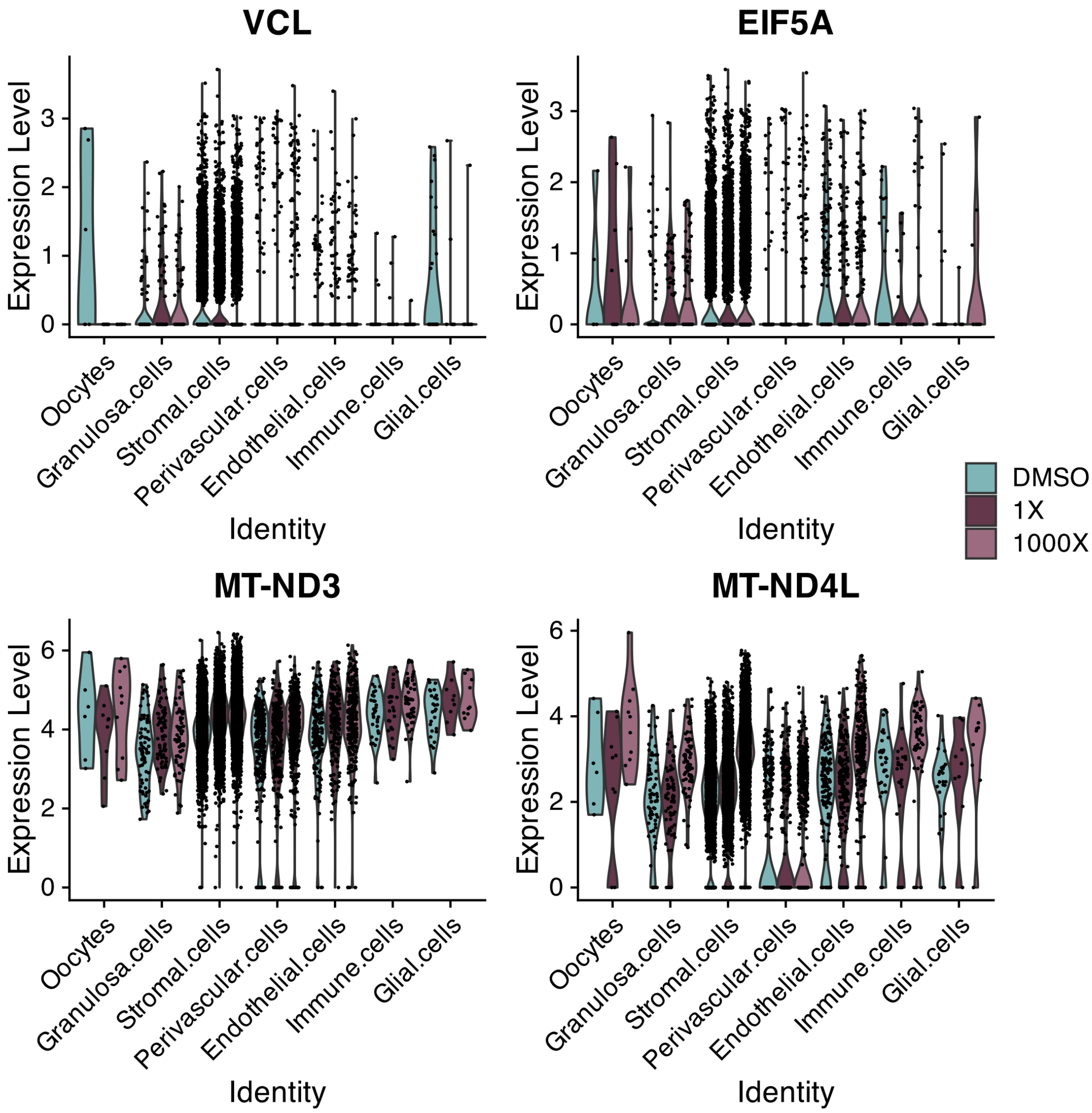


**Supplementary Figure 2. Expression of VCL, EIF5A, MT-ND3 and MT-ND4L by cell type in MEHP-treated human ovarian explants.** Violin plots show normalized expression levels of each gene across cell types, split by treatment condition (DMSO, 1X, 1000X) and plotted side by side. Colors correspond to the dilution conditions, and individual dots represent single cells. Gene expression values were log-normalized and visualized using the VlnPlot function from the Seurat package.

## Supplementary Figure 3

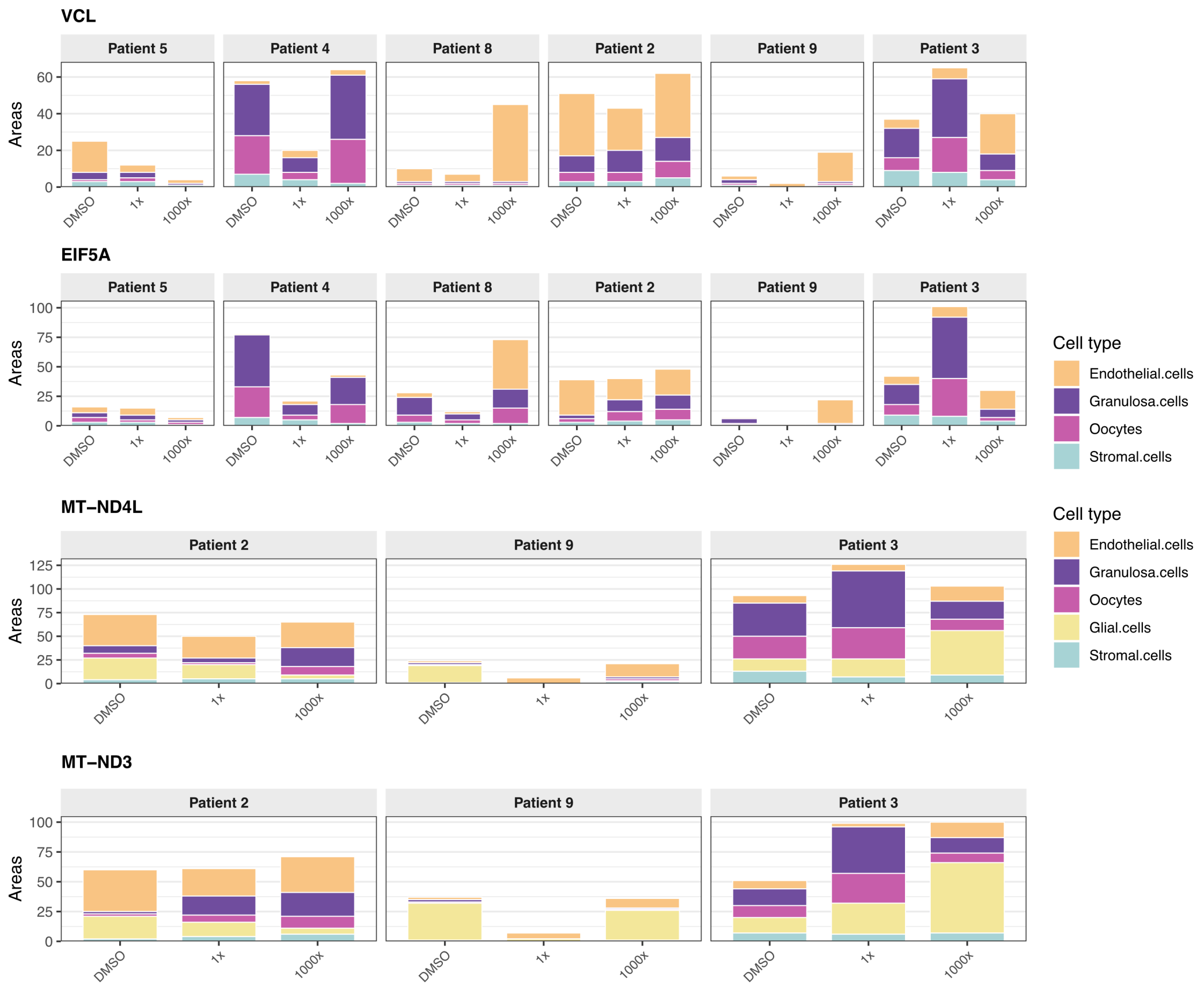


**Supplementary Figure 3. Number of data points per patient used for quantification of immunofluorescence images.**

## Supplementary Figure 4

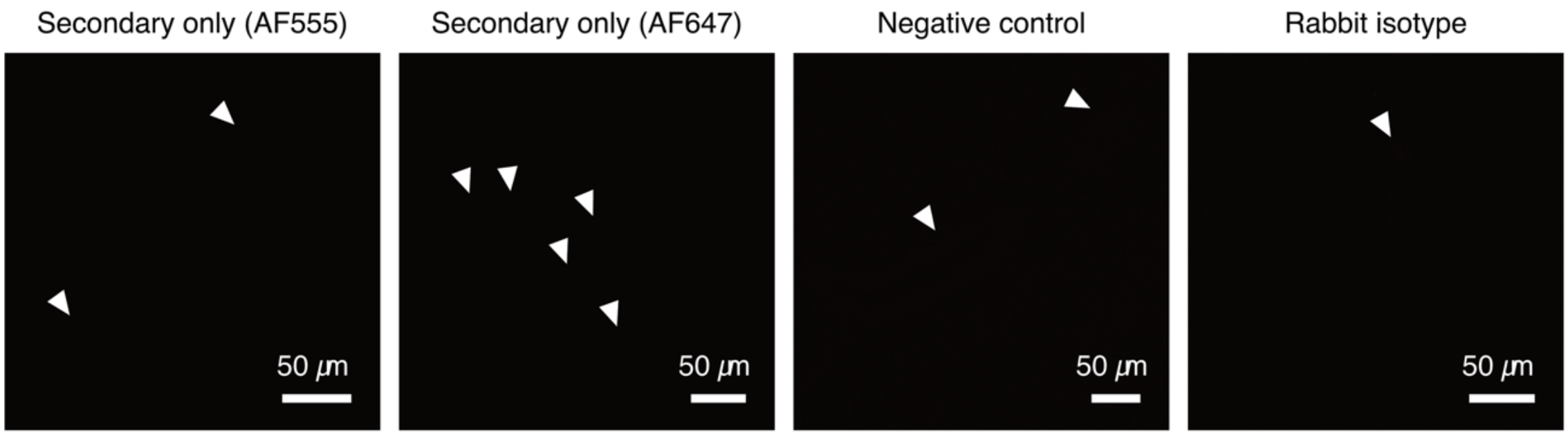


**Supplementary Figure 4. Negative controls of immunofluorescence staining on human ovarian cortical tissue exposed to DMSO for 6 days.**

The first two images show secondary antibody–only controls using Alexa Fluor (AF) 555 or AF 647, each imaged in its respective single channel (red or far-red). The third panel shows the negative control (no antibodies), and the fourth shows the rabbit isotype control, both imaged using settings for AF 555 and AF 647. Arrowheads indicate blood vessels. For each control, the imaging settings corresponding to the highest brightness and contrast used for the target-stained samples were applied to confirm the absence of non-specific signal. The negative controls confirmed the absence of non-specific antibody binding and tissue autofluorescence. Figures were made using omero.figure.

# Supplementary Figure 5

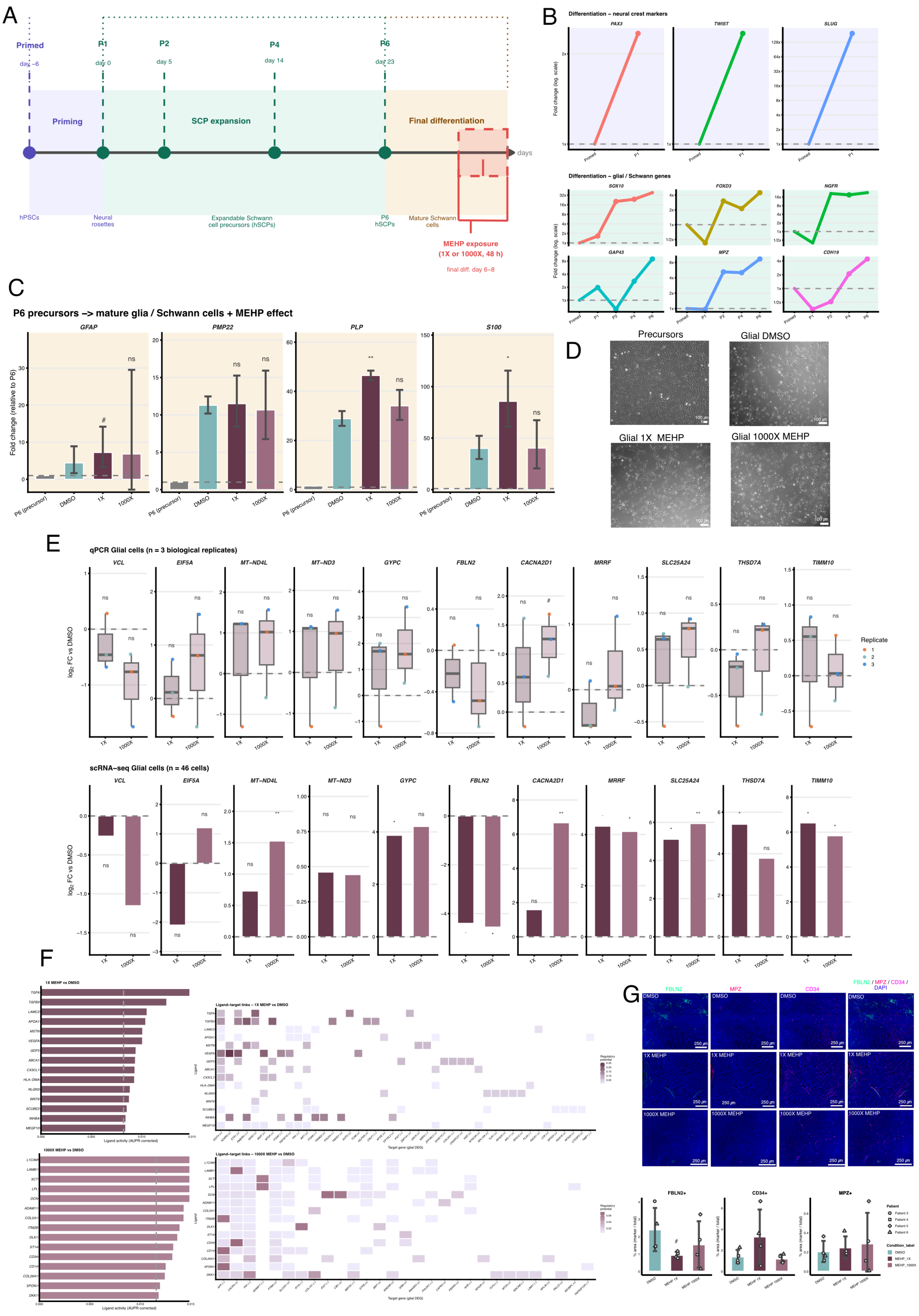

**Supplementary Figure 5. Differentiation pipeline of glial/Schwann cells from human induced pluripotent stem cells.**

(A) Outline of differentiation pipeline of glial/Schwann cells.
(B) Gene expression changes of neural crest and glial cell markers compared to primed stem cells.
(C) Glial cell marker expression after maturation of precursor cells and the effect of MEHP on marker expression.
(D) Representative images of precursor cells and mature glial cells under DMSO, 1× MEHP, and 1000× MEHP exposure conditions.
(E) Comparison of qPCR gene expression results with single-cell sequencing data.
(F) ENicheNet analysis of the glial cell cluster from single-cell RNA-seq data.
(G) Multiplex immunofluorescence staining of FBLN2, CD43, and MPZ in patient samples exposed to MEHP. Representative images from one patient are shown across the three staining channels. Quantification was performed using samples from four patients.

qPCR and multiplex results were analysed using a one-sample t-test on log2 fold changes. # $p < 0.1$. Single-cell gene expression differences were assessed using the Wilcoxon rank-sum test.
